# A critical overview of current progress for COVID-19: development of vaccines, antiviral drugs, and therapeutic antibodies

**DOI:** 10.1186/s12929-022-00852-9

**Published:** 2022-09-12

**Authors:** Monika Kumari, Ruei-Min Lu, Mu-Chun Li, Jhih-Liang Huang, Fu-Fei Hsu, Shih-Han Ko, Feng-Yi Ke, Shih-Chieh Su, Kang-Hao Liang, Joyce Pei-Yi Yuan, Hsiao-Ling Chiang, Cheng-Pu Sun, I.-Jung Lee, Wen-Shan Li, Hsing-Pang Hsieh, Mi-Hua Tao, Han-Chung Wu

**Affiliations:** 1 Biomedical Translation Research Center (BioTReC), Academia Sinica, Taipei, 11571 Taiwan; 2 Institute of Cellular and Organismic Biology, Academia Sinica, No. 128, Academia Road, Section 2, Nankang District, Taipei, 11529 Taiwan; 3 Institute of Chemistry, Academia Sinica, Taipei, 11529 Taiwan; 4 Institute of Biomedical Sciences, Academia Sinica, Taipei, 11529 Taiwan; 5 Institute of Biotechnology and Pharmaceutical Research, National Health Research Institutes, Miaoli County, 35053 Taiwan

**Keywords:** COVID-19, SARS-CoV-2, Therapeutics, mRNA vaccines, Small molecule antiviral drugs, Neutralizing antibodies, Vaccine development

## Abstract

The novel coronavirus disease (COVID-19) pandemic remains a global public health crisis, presenting a broad range of challenges. To help address some of the main problems, the scientific community has designed vaccines, diagnostic tools and therapeutics for severe acute respiratory syndrome coronavirus 2 (SARS-CoV-2) infection. The rapid pace of technology development, especially with regard to vaccines, represents a stunning and historic scientific achievement. Nevertheless, many challenges remain to be overcome, such as improving vaccine and drug treatment efficacies for emergent mutant strains of SARS-CoV-2. Outbreaks of more infectious variants continue to diminish the utility of available vaccines and drugs. Thus, the effectiveness of vaccines and drugs against the most current variants is a primary consideration in the continual analyses of clinical data that supports updated regulatory decisions. The first two vaccines granted Emergency Use Authorizations (EUAs), BNT162b2 and mRNA-1273, still show more than 60% protection efficacy against the most widespread current SARS-CoV-2 variant, Omicron. This variant carries more than 30 mutations in the spike protein, which has largely abrogated the neutralizing effects of therapeutic antibodies. Fortunately, some neutralizing antibodies and antiviral COVID-19 drugs treatments have shown continued clinical benefits. In this review, we provide a framework for understanding the ongoing development efforts for different types of vaccines and therapeutics, including small molecule and antibody drugs. The ripple effects of newly emergent variants, including updates to vaccines and drug repurposing efforts, are summarized. In addition, we summarize the clinical trials supporting the development and distribution of vaccines, small molecule drugs, and therapeutic antibodies with broad-spectrum activity against SARS-CoV-2 strains.

**Supplementary Information:**

The online version contains supplementary material available at 10.1186/s12929-022-00852-9.

## Introduction

**Fig. 1:**
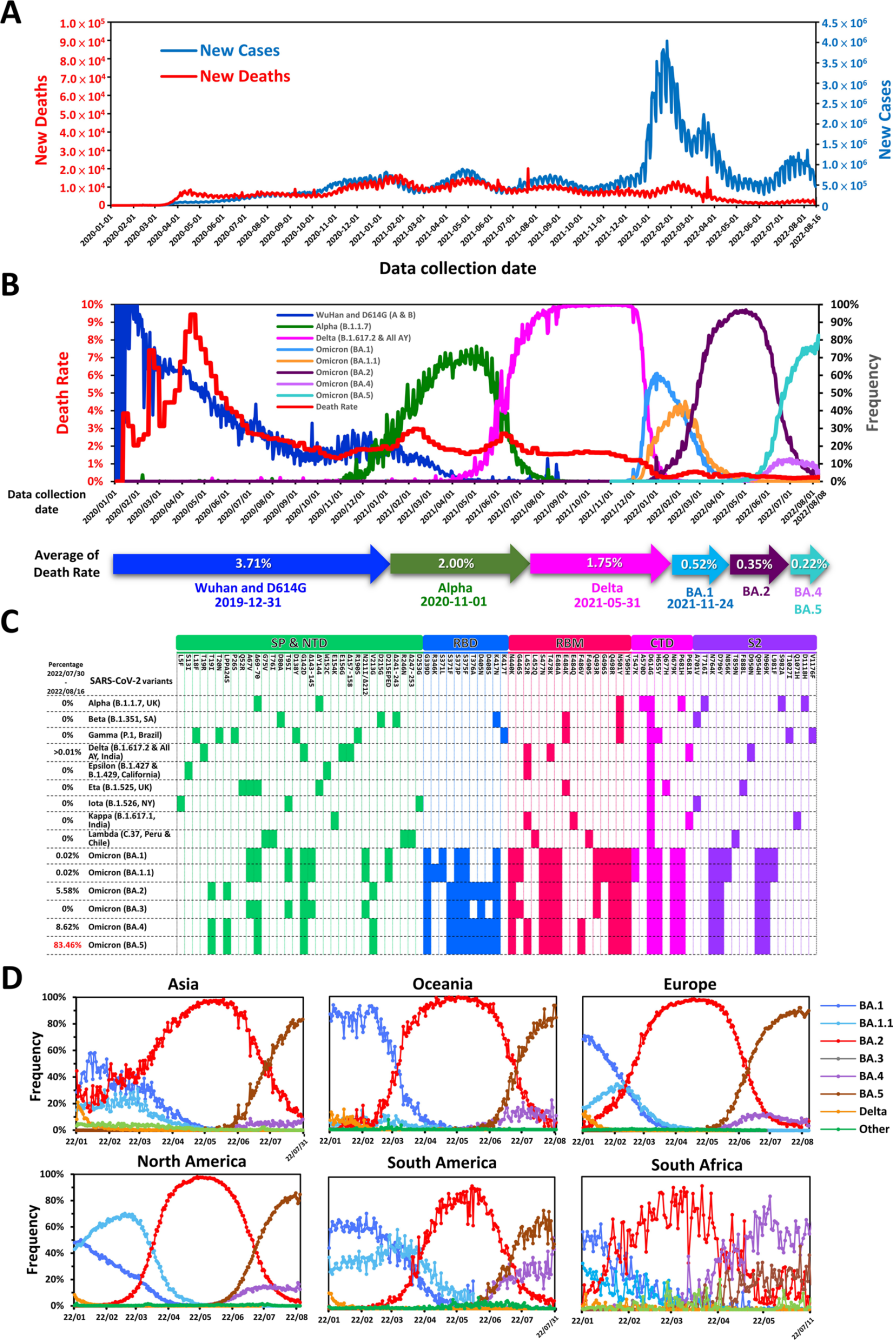
Global circumstances regarding COVID-19. **A** Resurgence of new cases is associated with increased mortality. Data were collected from the WHO COVID-19 Dashboard. Geneva: World Health Organization, 2020; available online: https://covid19.who.int/. **B** Epidemic dynamics of SARS-CoV-2 dominant variants. The data for frequency of infection by each variant were collected from GISAID [251]. The death rate was calculated as weekly deaths/weekly cases from (**A**). **C** Genomic variations in spike protein of major and emerging SARS-CoV-2 variants. **D** Epidemic dynamics of SARS-CoV-2 Omicron variants on five continents and South Africa; represented as daily frequency of each detected sequence. Due to a lack of sufficient data from South Africa in June July 2022 (daily sequencing cases < 15), the Omicron frequency analysis for South Africa was only performed up to July 11, 2022. All data were retrieved from GISAID

The first coronavirus disease 2019 (COVID-19) infections were reported in late-December 2019, and the disease spread rapidly around the world, echoing the fearsome global outbreak of “Spanish flu” 101 years prior [1]. As of August 18, 2022, there have been 595 million confirmed COVID-19 cases and more than 6.45 million deaths recorded globally (Fig. 1A) [2]. However, some countries tackle this chaotic situation better than others. Additional file 1: Table S1 shows the summarized data of COVID-19 confirmed total cases, deaths, and death rate in the selected developed countries. In recent decades, the strongest line of defense against pathogen outbreaks has been vaccines, which have greatly reduced the rates of morbidity and mortality from many deadly viruses and bacteria [3]. Over the years, several different approaches have been taken to design and develop vaccines against different viral infections. Currently available vaccines may be made from live attenuated virus, inactivated virus, purified antigen, or nucleic acids. Despite the options in vaccine design, the development of a vaccine usually takes many years to progress from the initial design stage to approval and clinical application. Moreover, before the COVID-19 pandemic, successful progression of a vaccine from preclinical studies to clinical trials only occurred for a very low percentage of candidates. One major factor impeding vaccine development is insufficient numbers of subject enrollments for testing. Therefore, companies focusing on vaccine development tend to test effectiveness and safety in animals rather than in humans [4]. The lack of appropriate safety data in humans markedly lowers the chance of success in clinical trials, and those vaccines that do progress to clinical trials typically exhibit poor balance between efficacy and safety.

In patients with COVID-19, the respiratory system may suffer major damage from dysregulated immune response associated with viral replication [5]. Recently finding suggest that the viral infection alters the gut microbes and the presence of viral RNA in the gut mucosa [6, 7]. Since severe disease is highly difficult to treat, the research community has prioritized the creation of prophylactic treatments. After the sequence of the viral genome was published, several types of vaccines against SARS-CoV-2 spike (S) protein were quickly developed and received Emergency Use Authorization (EUA) or approval in countries around the world (Table 1A). Among the vaccines successfully produced, those composed of lipid nanoparticles (LNPs) encapsulated messenger RNA (mRNA) have gained significant attention [8, 9]. As of September 6, 2022, 781 trials in 79 countries for SARS-CoV-2 vaccines had been initiated (https://covid19.trackvaccines.org/). Moreover, the enormous financial and health burden of COVID-19 has caused governments around the world to allow clinical trials to proceed faster and permit companies to run several tests concurrently (e.g., route of immunization, number of injections, and interval between doses).

**Table 1:** Different types of vaccine in clinical trials against COVID-19

A. The efficacies of WHO-approved COVID-19 vaccines						
Vaccine name	Brand name	Manufacturer	Platform	Doses administered*	Dose regimens	Vaccine efficacy% (95% CI)
BNT162b2	Comirnaty	Pfizer-BioNTech	mRNA	1,785,619,896	2 doses (21 days apart)	94.6% [124]
mRNA-1273	Spikevax	Moderna	mRNA	542,622,974	2 doses (28 days apart)	94.1% [125]
AZD1222	Vaxzevria	AstraZeneca-Oxford	Viral vector	200,591,659	2 doses (28 days apart)	66.7% 55.1% (2 doses < 6 weeks apart) 81.3% (2 doses > 12 weeks apart) [44]
	Covishield	Serum Institute of India	Viral vector	N.D	2 doses (4–8 weeks apart)	~ 90% [127]
Ad26.COV2.S	Janssen COVID-19 Vaccine	Johnson & Johnson	Viral vector	67,448,407	1 dose	66% [126]
BBIBP-CorV	Covilo	Sinopharm	Inactivated virus	73,451,148	2 doses (21 days apart)	79% [232]
COVID-19 Vaccine	CoronaVac	Sinovac Biotech	Inactivated virus	61,179,382	2 doses (14 days apart)	50.4% (Brazil), 67% (Chile), 65% (Indonesia), 78% (Brazil), 84% (Turkey) *Sinovac/CoronaVac COVID-19 vaccine*
BBV152	Covaxin	Bharat Biotech	Viral vector	N.D	2 doses (28 days apart)	81% [129]
NVX-CoV2373	Nuvaxoid	Novavax	Protein subunit	751,181	2 doses (21 days apart)	89.7% [61]
	Covovax	Serum Institute of India	Protein subunit	N.D	2 doses (21 days apart)	90.4% (USA) 89.7% (UK and Mexico) *COVOVAX (seruminstitute.com)*

B. COVID-19 vaccines in Phase III trial against different variants				
Vaccine name	Manufacturer	Types/Targets	Country performing Clinical trial	Trial ID
mRNA-1273.213	Moderna	N1-Methyl-pseudouridine (N1mΨ) synthetic mRNA / Omicron	USA	NCT04927065
BNT162b2s01	Pfizer-BioNTech	N1mΨ synthetic mRNA / Beta	Argentina, Brazil, Germany, South Africa, Turkey, and USA	NCT04368728
ARCT-154	Arcturus therapeutic	Self-amplifying mRNA / Alpha, Beta, Gamma and Delta	Viet Nam	NCT05012943
mRNA-1273.529	Moderna	N1mΨ synthetic mRNA / Omicron	United Kingdom and Northern Ireland	NCT05249829
AZD2816	AstraZeneca-Oxford	AZD2816 (ChAdOx1-vectored vaccine)/ Beta	Brazil, United Kingdom, and Northern Ireland	NCT04973449

^*^ Source: Hannah Ritchie, Edouard Mathieu, Lucas Rodés-Guirao, Cameron Appel, Charlie Giattino, Esteban Ortiz-Ospina, Joe Hasell, Bobbie Macdonald, Diana Beltekian and Max Roser (2020)—"Coronavirus Pandemic (COVID-19)". Published online at OurWorldInData.org. Retrieved from: 'https://ourworldindata.org/coronavirus' [Online Resource] Data extracted 04 May 2022

N.D., not determined

Despite the large number of COVID-19 vaccines in development and on the market, vaccine availability remains a major challenge throughout the world. Vaccination can reduce the number of infections and death rates, but the rapid emergence of viral variants such as Alpha, Beta, Gamma, Delta, and Omicron has jeopardized the efficacies of current vaccines and increased the urgency of making vaccines available worldwide (Fig. 1B–D).

The development of vaccines against COVID-19 represents a major breakthrough in the scientific world, though patients with high health risk (e.g., individuals who are immunocompromised or who have certain comorbidities) cannot rely on vaccines due to potentially severe side effects and low levels of antibody production. Instead, such patients require a range of therapeutic treatments that are appropriate for their disease severity. Passive immunity may be conferred to such patients by administering external neutralizing antibodies (nAbs) to treat and prevent viral infection; these nAbs act mainly through binding and neutralizing the virus. Using single human B cell antibody technology, nAbs against SARS-CoV-2 have been rapidly identified from convalescent patients. After identification, the nAbs may be evaluated in pre-clinical studies according to the US FDA-recommended accelerated phase I CMC monoclonal antibody (mAb) timeline [10]. Under these guidelines, the fastest development timeline for a nAb can be 5–6 months from the initial discovery to filing of the Investigational New Drug (IND) application. This accelerated program allowed the anti-SARS-CoV-2 human nAb, bamlanivimab, to become the first SARS-CoV-2-specific drug to receive an EUA from the US FDA on November 9, 2020 [11, 12]. Then on November 21, 2020, the REGEN-COV nAb cocktail (casirivimab and imdevimab) received an EUA for treatment of COVID-19 patients [13–16]. In the clinic, these nAbs provided immediate passive immunity to patients, drastically reducing viral and disease burden as well as breaking the chain of virus transmission. Meanwhile, small molecule antiviral drugs were generated to interfere with the virus life cycle and inhibit viral replication. On December 22, 2021, Paxlovid (nirmatrelvir and ritonavir) received an EUA that made it the first orally administered direct antiviral drug for SARS-CoV-2 treatment. One day later, the US FDA issued an EUA to another orally administered small molecule, Lagevrio (molnupiravir), for the treatment of patients with COVID-19.

In this manuscript, we review different strategies that have been used for COVID-19 prevention and therapy, and we provide updated information on vaccines and therapeutic drugs that have been approved, authorized for emergency use, or are under clinical development.

## Prevention for COVID-19

**Fig. 2:**
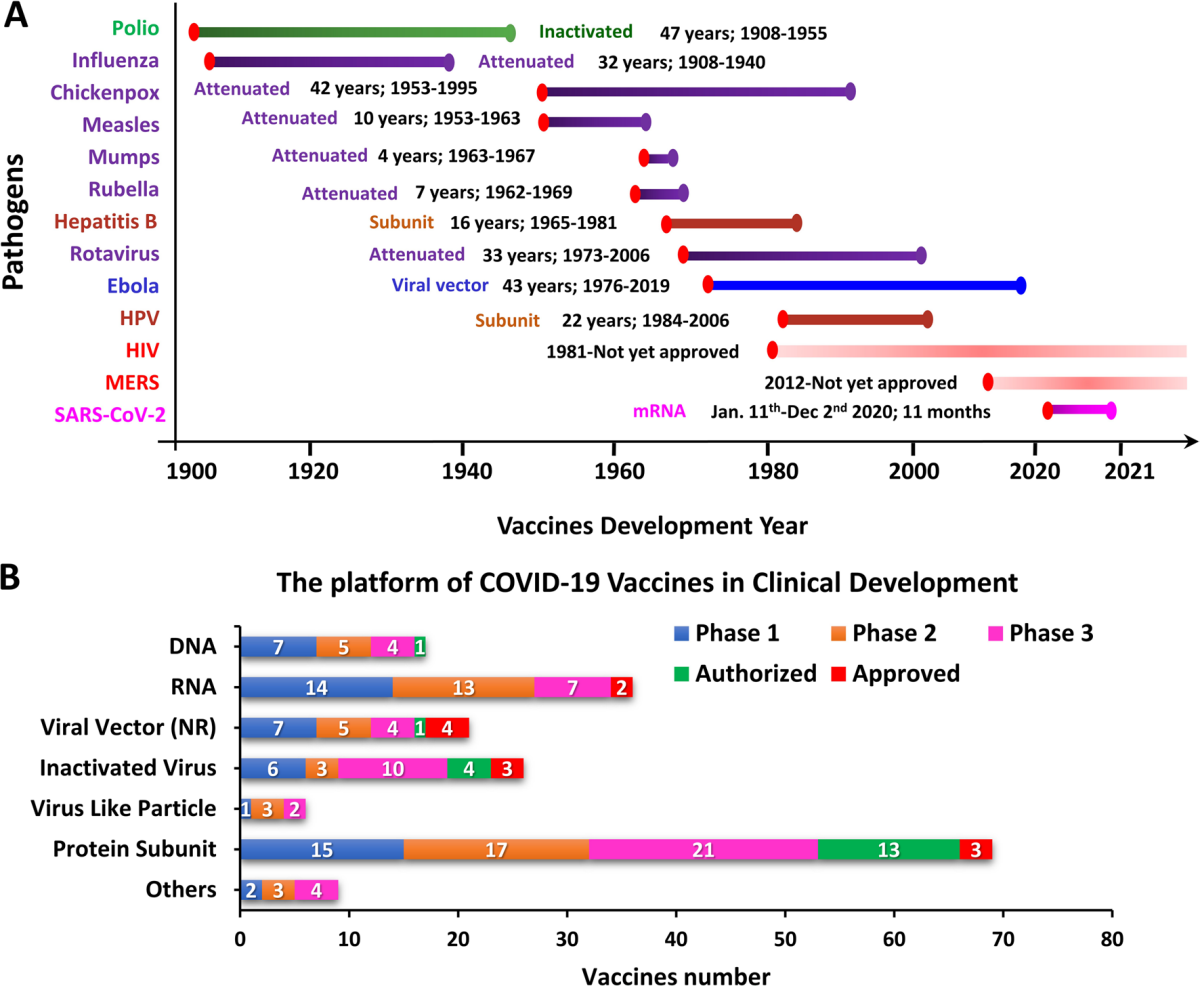
Global approaches in vaccines development. **A** Timeline of different vaccine development platforms against viral infections. The timeline represents the first vaccine developed against each pathogen outbreak. Color of the bar represents the vaccine type. Red dots indicate the years in which the pathogen was linked to diseases. **B** Number of candidate vaccines against SARS-CoV-2 of each vaccine platform type in various clinical stages. Data is acquired from COVID-19 vaccine tracker and landscape published by World Health Organization dated April 22, 2022. Viral vector (NR) indicates non-replicating viral vector; others include replicating viral vector, live attenuated virus, replicating viral vector plus antigen presenting cells, and non-replicating viral vector plus antigen presenting cells

The SARS-CoV-2 pandemic was first reported in Wuhan, China. Since then, a number of preventative efforts have been undertaken to minimize the virus spread. These include strict border controls, maintaining a social distance from others, wearing medical face masks, and isolation of patients with suspected infection or quarantine after close contact with infected individuals. The profound health and economic losses suffered by many countries have prompted governments to urge researchers from academic, biotech and pharmaceutical fields to devote themselves to developing diagnostics, vaccines and therapeutics that may be used to fight against the pandemic. Vaccines have played an integral role in reducing the spread of countless infectious diseases and some vaccines, for instance smallpox vaccines virtually, had made the disease globally eradicated. In 1980, the WHO announced routine smallpox vaccination is not required anymore [17]. Despite remarkable achievements such as this, vaccine development and production scale-up remain major challenges in a time of crisis. The process usually takes many years before final approval is granted and a product can enter the marketplace (Fig. 2A). Vaccine development also requires millions of dollars to bring a single product from the bench to the market [9, 18]. The quickest vaccine development timeline before the onset of COVID-19 was for the mumps vaccine, which took four years from development to deployment. The technological breakthrough that allowed such quick development was the use of attenuated virus [19]. A historical account of vaccine development timelines for different viral infections is shown in Fig. 2A.

### Global development of COVID-19 vaccines

To protect people from SARS-CoV-2 infection, tremendous research efforts have been made toward COVID-19 vaccine development. At least 198 vaccines are in pre-clinical development stages and 171 candidate vaccines have reached clinical trials [20]. Approaches to vaccine development have included protein subunits, nucleic acids (RNA and DNA), viral vectors (non-replicating and replicating), viruses (live attenuated and inactivated), and virus-like particles [21] (Fig. 2B). The vaccines that have gained approval from the World Health Organization (WHO) were developed based on a variety of approaches and have shown different levels of efficacy (summarized in Table 1A). As the S protein of SARS-CoV-2 plays an important role in receptor binding and membrane fusion, full-length S protein or its key fragments, such as its receptor binding domain (RBD), have been utilized as the main target antigen for protein-, nucleic acid- and vector-based vaccine candidates [22]. However, new strains of SARS-CoV-2 carry mutations in these antigens. The S protein mutations and global dynamics of new SARS-CoV-2 strains are shown in Fig. 1B–D. Twelve vaccine candidates in clinical development have so far been approved for use by different national regulatory agencies. Among the types of candidates, protein-based vaccines comprise the majority (32%), with 21 candidate vaccines number in Phase III and one in Phase IV [20]. NVX-CoV2373 from Novavax was the first protein-based vaccine to be approved by the European Medicine Agency (EMA). This product was approved in December 2021 for prevention of SARS-CoV-2 with an efficacy of 89.7% [23]. Phase II study results for the recombinant protein vaccine (MVC-COV1901) from Medigen Vaccine Biologics (Taipei, Taiwan) showed that participants who received MVC-COV1901 had anti-spike IgG GMT of 524.0 BAU/ml on day 57. Using a BAU (Binding Antibody Unit) model, it was predicted that vaccine efficacy should be in the range of 80–90% [24]. MVC-COV1901 is the first COVID-19 vaccine developed in Taiwan to acquire EUA from the Taiwan Food and Drug Administration [25]. The Phase IV study for this product is ongoing (NCT05097053 and NCT05079633).

The second largest group of vaccines in development, accounting for 24% of the total, are RNA-based vaccines [20] (Fig. 2B). One such vaccine, BNT162b2 from Pfizer-BioNTech, was the first to receive authorization from the WHO for emergency use to prevent COVID-19 [26]. Remarkably, mRNA-1273 from Moderna began its first US clinical trial just 66 days after the SARS-CoV-2 sequence was made available. These two products are the first RNA vaccines approved for clinical use and have clearly demonstrated that RNA-based vaccines offer several major competitive advantages and potential applications [27].

In the COVID-19 vaccine race, teams from 79 countries have performed clinical studies on 222 vaccine candidates, and more than two-thirds of the candidates have entered Phase II trials. Among the different types of vaccines tested in clinical studies, protein subunit vaccines remain the predominant type (32% of all candidates), followed by RNA (24%), inactivated virus (13%), non-replicating viral vectors (13%), and DNA (9%) [20].

Since the beginning of the pandemic, much has been learned about the different types of vaccines and their efficacy and safety. Currently, a major issue with vaccine use is equitable access to effective vaccines. Widespread distribution of vaccines that can effectively elicit an immune response to neutralize the SARS-CoV-2 infection will be a critical step in ending the COVID-19 pandemic. Among the extraordinary number of vaccines that are currently under development, nucleic acid-based vaccines have shown tremendous potential and emerged as viable alternatives to traditional vaccines. In the following sections, we discuss the rationale and design of three major types of vaccines (adenoviral vector, protein subunit, and mRNA), with some depiction on the design of lipid nanoparticles, the effectiveness of vaccines against different SARS-CoV-2 variants, and the suitability of vaccines for patients suffering from impaired immunity.

#### Adenoviral vector-based COVID-19 vaccines

Adenoviruses are by far the most common viral vectors used for SARS-CoV-2 vaccines. These DNA viruses are composed of a non-enveloped icosahedral capsid of approximately 90 nm in diameter and were first discovered in the 1950s [28]. Although most adenovirus infections are mild or asymptomatic, they can occasionally result in severe or life-threatening manifestations, particularly in immunocompromised persons [29]. The advantage of adenovirus-mediated broad gene expression was utilized in 1990s for therapeutic gene delivery to treat alpha-1 antitrypsin deficiency [30] and cystic fibrosis [31]. However, although adenoviral vectors deliver the genes of interest into host cells efficiently, host immune responses are also triggered, limiting vector transduction and transgene expression. The primary innate immunity induction of adenoviral vector is the viral genome which is sensed through cytosolic DNA sensors such as toll-like receptor 9 (TLR9) and cyclic guanosine monophosphate-AMP synthase (cGAS) and subsequently leads to production of pro-inflammatory chemokines and cytokines. The stimulatory markers on antigen presenting cells (APCs) can also be upregulated by simultaneous expression of antigens in this inflammatory environment. Consequently, APCs drive the maturation and expansion of cognate T and B cells which are critical for viral clearance and antibody production [32]. The transient gene expression and high immunogenicity of adenoviral vector make it an ideal vaccine platform requiring no additional adjuvants. After the SARS-CoV-2 genome sequence was unraveled in January 2020, adenoviral vector based-vaccine was selected as candidate of vaccine platform due to its manufacturing ease and rapid development as compared to protein or subunit vaccines. Up to now, four adenoviral vector-based vaccines have been approved by different regional authorities.

Human adenovirus 5 (Ad5) was originally the most common adenovirus vector to be utilized for vaccine development. The Ad5 vector-based COVID-19 vaccine, Ad5-nCoV, encodes a full-length mammalian-cell-optimized S protein with a tissue plasminogen activator (tPA) signal peptide; this vaccine was developed by CanSino and has been approved in China [33]. In Phase I and II clinical studies, Ad5-nCoV was well-tolerated and generated robust T cell and antibody responses [34, 35]. The efficacy of one dose of Ad5-nCoV was estimated to be 57.5% against symptomatic COVID-19 infection [36]. However, previous studies of the Ad5-based HIV and Ebola vaccine showed that the antigen-specific immune response could be attenuated by preexisting immunity to Ad5 [37, 38]. Therefore, the development of adenovirus-vector vaccines has trended toward use of a less seroprevalent human adenovirus (Ad26) [1] or non-human primate adenovirus (chAd) [39].

ChAdOx1 is a serotype Y25 chimpanzee adenovirus vector with additional modifications that substitute E4 regions with that of Ad5 to increase virus yields [40]. The ChAdOx1 nCoV-19 (AZD1222; brand name, Vaxzevria) vaccine from AstraZeneca was first granted conditional authorization for emergency use by the European Medicines Agency (EMA). AZD1222 expresses S protein with a tPA leader peptide as an antigen. In non-human primates challenged with SARS-CoV-2, a single vaccination of AZD1222 vaccine efficiently ameliorated pulmonary damage, and a prime-boost vaccination strategy further increased nAb titers [41]. The Phase I/II trial of AZD1222 vaccine adopted the prime-boost regimen and showed that the vaccine was well-tolerated and immunogenic, generating both nAb and T cell responses [41, 42]. In two Phase III trials, the overall vaccine efficacy in individuals receiving two standard doses was reported to be ~ 70% [43, 44]. Importantly, AZD1222-induced antibodies can facilitate antibody-dependent neutrophil/monocyte phagocytosis, complement deposition and NK cell activation [45], which may effectively control SARS-CoV-2 infection.

The Janssen COVID-19 vaccine (Ad26.COV2.S) expresses an engineered S protein that is stabilized by deletion of the furin cleavage site and two consecutive proline mutations [46, 47]; the product was first authorized by the U.S. Food and Drug Administration (FDA). A single shot of Janssen COVID-19 vaccine induced nAb responses, which was highly correlated with protection against SARS-CoV-2 challenge in non-human primates [47]. Phase I/II trials were initiated in July 2020 and showed good tolerability and immunogenicity [48]. In a Phase III trial, a single administration of the Janssen COVID-19 vaccine was found to be 66.9% effective against COVID-19 and provide higher protection (76.7%) against severe-to-critical symptoms at 14 days post-vaccination [49]. Similar to AZD1222, an Fc-mediated enhancement of innate immune response was also observed [50].

In another instance, the Russian Sputnik V vaccine was developed by the Gamaleya Research Institute using a heterologous prime-boost strategy of Ad26 and Ad5 (each encoding full-length S protein). In a Phase I/II clinical study, this vaccine was demonstrated to be safe and immunogenic, stimulating both cellular and humoral immune responses [51]. The interim analysis of the Phase III clinical trial in Russia demonstrated 91.6% efficacy against COVID-19 [52].

#### Protein subunit vaccines

Instead of administering the entire pathogen, protein subunit vaccines elicit immune responses to one or more purified viral protein. The antigens are commonly expressed in eukaryotic cells using different expression systems and formulated with different adjuvants. This strategy is considered a safe and reliable method, as the vaccine has no live components; thus, the possibility of pathogenicity is eliminated, and the vaccines can even be used in immunocompromised patients [53]. Additionally, subunit vaccines are a well-established technology that has been used for decades, and the products are relatively stable during storage and transport. However, the ability of protein subunit vaccines to trigger immune response is often low and may require adjuvants and multiple doses to elicit protective immune responses [54]. The development and manufacturing process of recombinant proteins is also time consuming and complicated. Vaccines for hepatitis B, human papillomavirus, and influenza are prominent examples of the many protein subunit vaccines approved for clinical use [55–57].

To develop SARS-CoV-2 protein subunit vaccines, full-length S protein or its antigenic fragments, such as the S1 subunit and RBD, most often serve as the antigen targets [58]. As of September 6, 2022, 17 protein subunit vaccines against SARS-CoV-2 have been approved for emergency use and 55 candidates are in clinical trials (https://covid19.trackvaccines.org/vaccines/approved/). Among these, NVX-CoV2373 is considered to be one of the leading protein subunit vaccines for SARS-CoV-2 and has been approved in 37 countries. It is comprised of recombinant full-length S protein expressed in a baculovirus-Sf9 system as the antigen and Matrix‐M as the adjuvant [59]. The recombinant S protein is stabilized in the prefusion conformation by the introduction of two proline residues at K986 and V987; the antigen is further rendered protease resistant by replacing RRAR with QQAQ at the S1/S2 polybasic cleavage site. The NVX-CoV2373 nanoparticle is formed by insertion of the purified S protein transmembrane domain into micellar cores of polysorbate 80, which presents multi-trimer rosettes [60]. Phase III clinical trials showed that a two-dose regimen of the NVX-CoV2373 vaccine conferred 89.7% protection against SARS-CoV-2 infection and had high efficacy against the B.1.1.7 variant [61].

#### mRNA-based vaccines

The emergence of the COVID-19 pandemic pushed the scientific community to develop vaccines without compromising safety and effectiveness in timelines as short as a few months. mRNA-based vaccines are undoubtedly the most popular choice for quick development because of the simple, yet robust and flexible technical strategy used to produce new candidate vaccines. This method has so far outcompeted the more tedious conventional methods of vaccine development and made it possible for COVID-19 vaccines to be created and tested within only a few months. Furthermore, mRNA vaccines exhibit good safety potential due to the non-infectious and non-integrating nature of the formulation; this class is also highly effective because of its rapid uptake and expression. Perhaps the most important advantage of mRNA vaccines is their cost-effective production [62]. In this section, we discuss technical aspects of constructing an mRNA-based vaccine.

### mRNA synthesis and modification

**Fig. 3:**
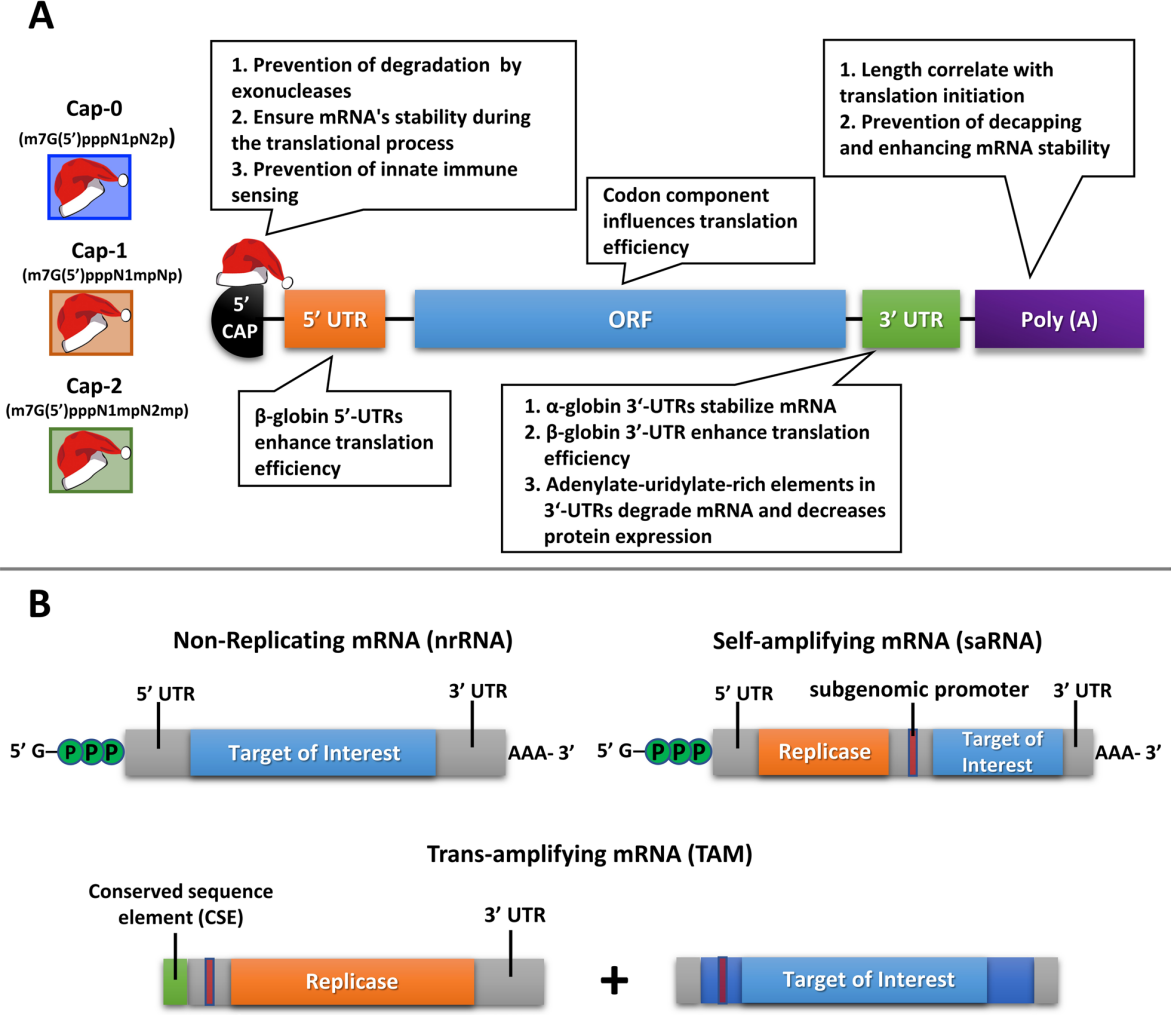
Schematic representation of the structure of conventional mRNA and the structure and intracellular amplification of self-amplifying mRNA. **A** The design of IVT mRNA is based on the blueprint of eukaryotic mRNA, and it consists of a 5’ cap, 5’ and 3’ untranslated regions (UTRs), an open reading frame (ORF) encoding antigen(s), and a 3’ poly(A) tail. The IVT mRNA can be modified in one or multiple sites, e.g., by modification of the caps, the UTRs and the poly(A) tail, to modulate the duration and kinetic profile of protein expression. **B** Antigen expression in different types of mRNA vaccines. The immunogen is encoded by a non-replicating RNA flanked by 5′ and 3′ UTRs. Self-amplifying RNA encodes four nonstructural proteins and a sub-genomic promoter derived from the alphavirus genome. It encodes a replicase and amplifies vaccine-antigen transcripts. Trans-amplifying RNA uses two transcripts to enable self-amplification of replicase and the target antigen

mRNA was first discovered in 1961 [63, 64]. Since then, numerous methods have been established to generate stable mRNA and protect it from degradation in a normal physiological environment. Because of its instability and low capacity to drive protein expression, mRNA was largely ignored as a drug modality after its discovery. To resolve the issue of instability in the body, scientists first modified mRNA structural elements, including 5′ and 3′ untranslated regions (UTRs) [65], poly(A) tail [66], 5′ cap [67, 68], and open reading frame (ORF) [69]. Each of these additions made significant improvements to the stability of synthetic mRNAs (Fig. 3A). Then, in 1984, Melton et al. introduced a method for in vitro transcription (IVT) of functional mRNA in cell-free system [70].

**Fig. 4:**
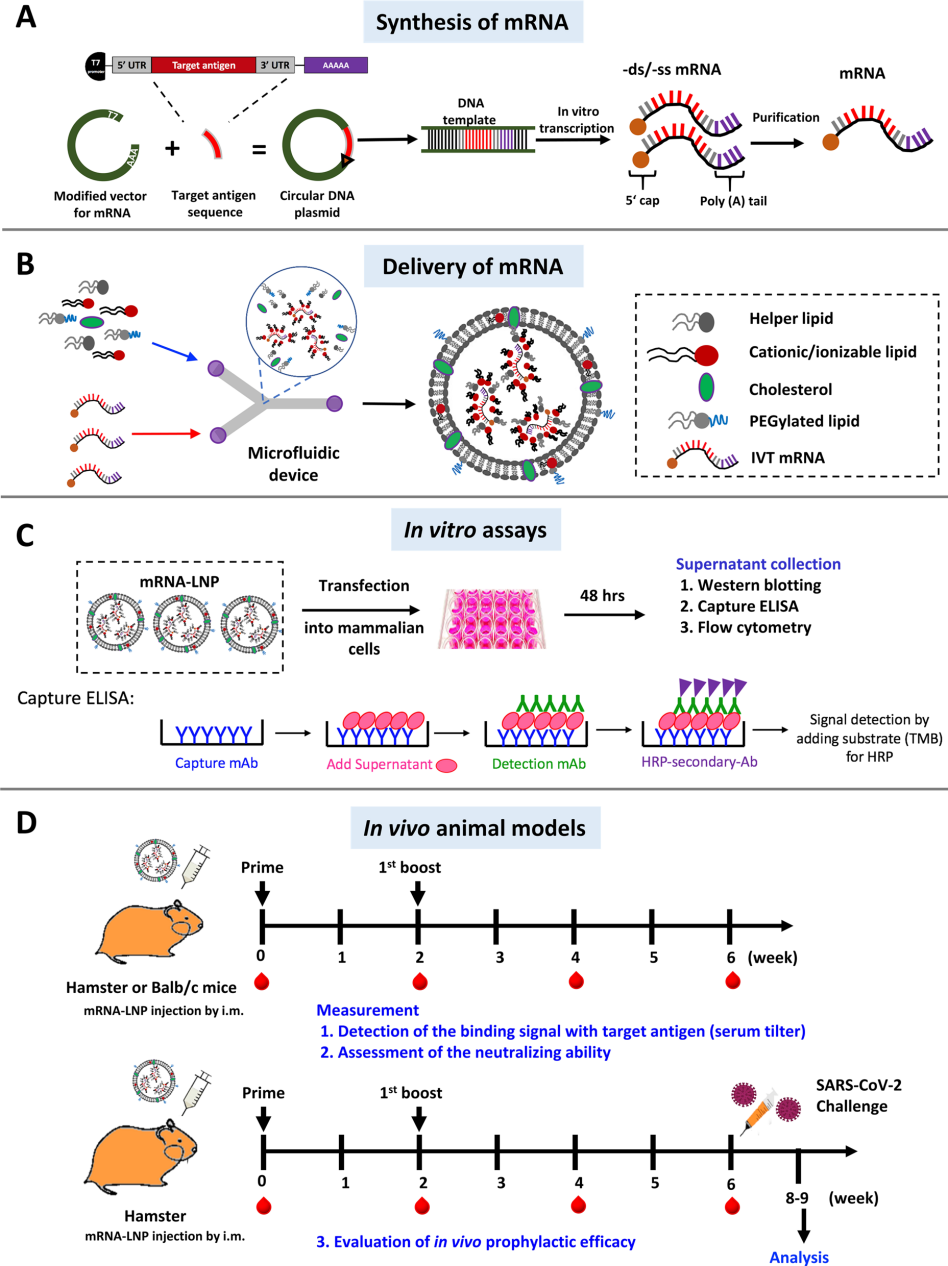
Diagrammatic illustration of mRNA-LNPs complex preparation and testing. **A** Synthesis of IVT mRNA. 1. Restriction enzyme digestion for DNA plasmid linearization; 2. Co-transcriptional capping of IVT; 3. DNase treatment and cellulose-based purification of IVT mRNA. **B** Schematic representation of the LNPs-encapsulated mRNA. **C** In vitro assay of protein expression from mRNA-LNPs. **D** Immunogenicity assessment of mRNA-LNPs in vivo

Still, the use of mRNA suffered from limitations of low translation efficiency, short functional half-life, and rapid degradation by ribonuclease enzymes. To address the issue of translation efficiency, scientists developed procedures for codon optimization before mRNA production and purification. Replacement with rare codons led to higher levels of controllable protein translation [71]. For instance, mRNA with high guanine-cytosine ratio (G:C) along with 5´ and 3´ modifications showed higher levels of protein expression [72]. In addition, the secondary structure of mRNA plays a major role in determining the ribosome dwelling time and mRNA half-life; it can also be manipulated to improve mRNA resistance to cleavage by endonucleases and chemical degradation processes [73]. mRNA capping is essential in the creation of stable and mature mRNA able to undergo translation during protein synthesis. The 5’ cap structure (m7GpppN) is a typical characteristic of eukaryotic mRNAs. It is composed of an N7-methylated guanosine linked to the first nucleotide of the RNA via a reverse 5’ to 5’ triphosphate bridge structure called Cap-0. In humans, the Cap-0 structure is further modified to a Cap-1 or a Cap-2 structure by respective 2’-O-methylation on the first or both nucleotide riboses (Fig. 3A). Incorporation of modified nucleotides, such as pseudouridine, 2-thiouridine, 5-methyluridine, 5-methylcytidine, or N6-methyladenosine, during IVT has been further shown to extend mRNA stability and modulate immune-stimulatory activity [74]. For instance, 1-methyl-pseudouridine (1mΨ) can drastically affect the secondary structure of mRNA to enhance certain mRNA structural characteristics and translation efficiency. 1mΨ also decreases the immunogenicity of mRNA and increases its thermostability and biological stability [73]. Furthermore, mRNA produced by IVT may contain impurities, such as double-stranded and/or broken fragments. HPLC or FPLC purification of mRNA can therefore reduce its immunogenicity and enhance the mRNA quality [75]. Figures 3A and 4A summarize current design principles and process for mRNA production by IVT. This modified nucleoside technology was licensed to both Moderna and Pfizer-BioNTech and was key to the success of COVID-19 mRNA vaccines [27].

Moderna’s mRNA-1273 and Pfizer-BioNTech BNT162b2 utilize different heavily modified mRNA sequences to induce stable and abundant target protein expression. Both technologies incorporate modified sequences around the start codon, using the sequence GCCACC**AUG** instead of GCCRCC**AUG**G. Elimination of the R and G residues at the 4^th^ and 10^th^ positions enhance translational initiation at a downstream AUG start codon. Following the start codon, the mRNA in BNT162b2 contains a small flanking region with secondary structure while the mRNA-1273 mRNA exhibits a much more pronounced secondary structure [76]. The 5′ UTR of mRNA-1273 is rich in GC content, while the 5′ UTR of BNT162b2 is derived from the human α-globin (HBA1) gene. Both vaccines encode the original S protein of SARS-CoV-2. However, the S protein gene in mRNA-1273 has all GAA codons replaced with GAG, while the 14 GAA codons of BNT162b2 remain unchanged [77]. Both mRNA vaccines utilize incorporation of 1mΨ [8]. Moreover, the amount of mRNA delivered in the BNT162b2 vaccine (30 μg/dose) is relatively lower than that in the mRNA-1273 product (100 μg/dose). The higher doses of mRNA-1273 might reflect the pronounced secondary structure and richness in GC content, which can both decrease translation initiation efficiency and protein expression.

### Type of synthetic mRNA

Two major types of mRNA have been extensively studied for in vivo protein expression applications: non-replicating mRNA (nrRNA) and self-amplifying mRNA (saRNA) (Fig. 3B). As discussed above, the non-replicating type has been used in conventional mRNA vaccines. However, vaccines made with nrRNA suffer from several serious limitations, such as a requirement for storage at low temperatures, poor stability, and unwanted side effects caused by large doses of mRNA [78, 79]. Researchers are continually making efforts to improve mRNA vaccines, finding ways to cut costs and increase the availability of vaccines worldwide. A major concern about this type of vaccine is the potential side effects that may arise with multiple doses. Additionally, a multiple dosing regimen requires a large manufacturing unit to create large amounts of vaccine. Therefore, scientists are currently working to develop protocols for synthesizing mRNAs that induce high-level protein expression. Such protocols may serve to minimize the number of doses. One recent approach is the use of saRNA. Unlike nrRNA, saRNA constructs encode four non-structural proteins comprising the replicase complex from alphavirus. This RNA-dependent RNA polymerase (RdRP) complex enables amplification of the mRNA in situ [80]. As a result of the self-replicative activity, higher expression levels of a vaccine antigen can be achieved with a relatively low mRNA dose. Thus, the saRNA approach may offer key advantages, such as reduced side effects, ease of optimization, and desirable manufacturability [81]. Vogel et al. showed that 64-fold less saRNA produced a similar level of protein expression in a trial influenza virus vaccine, as compared with an nrRNA-containing formulation. Moreover, the generation of double-stranded RNA intermediates during saRNA replication can provide additional immune stimulation [82] by activating interferon pathways, resulting in a self-adjuvant effect [83]. Despite these potential advantages, saRNA delivery still remains a major challenge, mainly because saRNA are larger due to the additional genes. Typically, saRNA contain 10,000 or more nucleotides (10 Kb), which is much larger than conventional nrRNAs of roughly 2000 nucleotides [78, 84]. The larger size makes both purification and production of saRNA challenging. Furthermore, encapsulation of larger mRNAs might reduce binding efficiency with non-viral vectors and make the formulations more difficult to deliver. Most importantly, a correct balance between saRNA-mediated protein expression and adequate immune stimulation will be needed for the best vaccine outcome. Currently, scientists are trying to improve saRNA delivery systems by introducing on/off synthetic RNA circuits, which might allow for controlled expression of immunomodulators [85]. Another approach is the introduction of trans-amplifying mRNAs; in this modality, the saRNA is divided into two transcripts, thus reducing the sizes of individual saRNAs (Fig. 3B). Beissert et al. has demonstrated the feasibility of using a trans-replicon system by generating a trans-replicon vaccine against influenza virus [86]. Notably, a SARS-CoV-2 saRNA called ARCT-154 is being evaluated in a recently initiated Phase III clinical trial in Vietnam (NCT05012943).

### mRNA delivery systems

Upon in vivo delivery, naked mRNAs will be rapidly degraded by extracellular ribonucleases. Therefore, complexing agents that stabilize the mRNAs play a significant role in the success of mRNA vaccines. A good complexing agent should enhance the cellular uptake and allow the mRNA to escape the endo-lysosomal compartment without causing cytotoxicity [79, 87]. The most popular delivery systems are composed of cationic polymers, cationic lipids, or peptides/proteins. Meanwhile, recent breakthroughs in LNPs have come from the incorporation of ionizable lipid technologies and microfluidic devices (Fig. 4B) [88].

In Fig. 4C, D we show schematic representations of an in vitro test for mRNA-LNPs-mediated protein expression and an in vivo test for mRNA-LNPs-mediated immunogenicity. As mRNA-LNPs can stimulate immunogenic protein translation in vivo, this technology is a versatile tool that may be used in several applications. Aside from vaccine development, mRNA-LNPs are also useful for generation of mAbs to treat emerging infectious diseases [89, 90], CAR-T cell therapy [91], gene editing [92], and RNA-based protein replacement therapies (RPRTs) [93].

### Cationic or ionizable lipids in lipid nanoparticles (LNPs) design

**Fig. 5:**
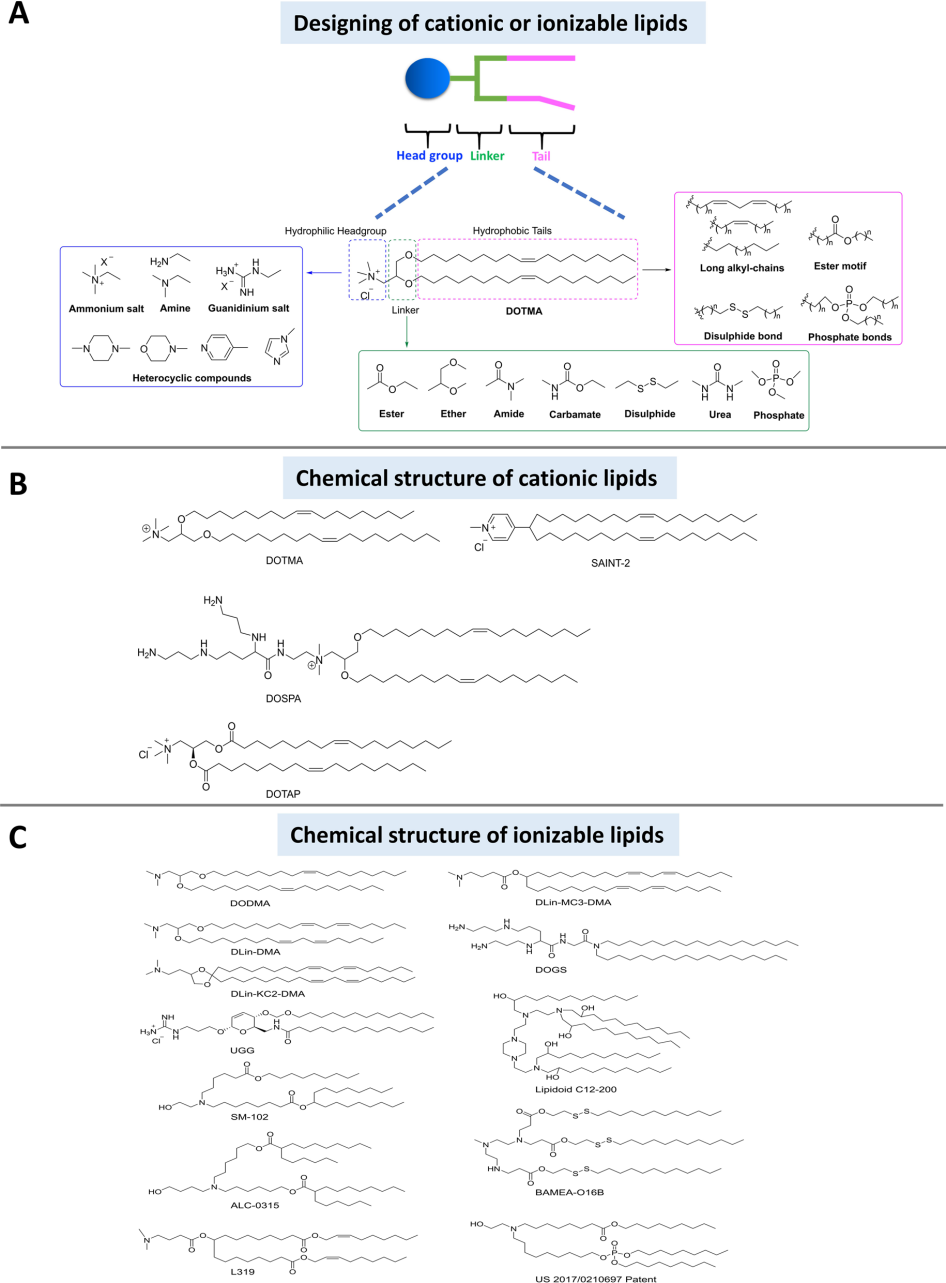
Chemical structure of most common lipids for mRNA delivery. **A** Cationic or ionizable lipid design. Analysis and summary of the representative structure of **B** Cationic lipids and **C** Ionizable lipids

In this section, recent progress in the design of cationic and ionizable lipids and their functions relating to mRNA-LNPs formulations is described. mRNA-LNPs mainly consist of four components in addition to mRNA [94]: (1) cationic or ionizable lipids with positive charges that bind to negatively charged mRNA, (2) PEGylated lipids that coat the LNPs and stabilize mRNA, (3) phospholipids, and (4) cholesterol molecules that maintain structural integrity [95]. The cationic or ionizable lipids are amphiphilic molecules that typically feature a polar head group, a hydrophobic tail, and a heteroatom linker between the two components [96] (Fig. 5). Design of effective cationic or ionizable lipids can be accomplished by fine-tuning the structures of the polar head group, hydrophobic tail and heteroatom linker, which will modulate the behaviors of the resulting mRNA-LNPs complexes.

#### Polar head group

Most commonly, polar heads contain N or other heteroatoms, such as ammonium salts, amines, guanidinium salts or heterocyclic compounds [96]. The positive charge (pH < pKa) or high electronegativity on each of these moieties can promote complexation of nucleic acids through charge-charge interactions [97] or hydrogen bonding [98]. The polar head group also controls release of the mRNA from endosomes (i.e., gene transfer efficiency) through a proton sponge effect [99]. Moreover, the dimension and charge density of the polar head group is critical for lipoplex stability, cell membrane interactions, endosomal escape, and mRNA compaction in the mRNA-LNPs [100]. Of late, multivalent head groups on cationic lipids have attracted great attention, as these head groups enhance binding with nucleic acids, segregate the complex from the intracellular environment, and increase transfection efficiency as compared to monovalent head groups [101].

Notably, quaternary ammonium (NR_4_^+^) head groups bearing hydroxyl groups have not been widely applied due to their uncertain effectiveness at promoting nucleic acid release and stability of complexes [102, 103]. Nevertheless, primary amines, secondary amines, and tertiary amines in head groups have been widely investigated in terms of acid–base properties. The pKa values of these primary, secondary, and tertiary amines, are 10.6, 10.8, and 9.8 respectively, and they display a long half-life in the body. A recent study also showed that head groups bearing tertiary amines serve to increase transfection efficiency by conferring the lipids with buffering capacity that expedites endo/lysosome escape and mRNA release within the cells [104, 105]. Guanidine is also sometimes used as a head group for ionizable lipids due to its delocalized charge across three N atoms and strong nucleic acid binding properties. However, its tight binding with nucleic acids has been shown to reduce the effectiveness of gene delivery [106]. Heterocyclic head groups, such as pyridine, imidazole, melamine and others, are frequently utilized in ionizable lipids due to their abilities to act as both an acid and a base. Several reports have demonstrated proton sponge effects and pH-sensitive functions of heterocyclic head groups, which serve to enhance transfection efficacy and endosomal escape [107, 108].

In summary, the polar head groups of cationic or ionizable lipids play important roles in gene delivery and transfection efficiency due to their participation in the initial mRNA binding and the ultimate mRNA release in the cytoplasm. As such, polar head groups with pKa > 7.4 are key components in the preliminary design of cationic or ionizable lipids.

#### Hydrophobic tail

The hydrophobic tail of cationic or ionizable lipids functions to modulate the phase transition, fluidity, stability, and cytotoxicity of mRNA-LNPs [109]. Usually, saturated or unsaturated aliphatic tails, such as stearyl or oleyl moieties, have been utilized. It is widely reported that the lipid chain number, length and degree of unsaturation all affect transfection efficacy. However, the relationship between length of the lipid and effectiveness of transfection remains a topic of debate. In general, there seems to be a consensus that either a hydrophobic tail with 10–14 carbon atoms confers the most effective in transfection efficiency [110] or a hydrophobic tail with a C14 displays optimum performance (C14 > C12 > C10 and C14 > C16 > C18) [111]. In addition, it is widely agreed that an asymmetric hydrophobic tail is highly recommended to increase transfection efficiency.

As shown in Fig. 5C, DODMA was one of the first ionizable lipids for gene delivery. Its single alkyl-chains were originally used in the design of ionizable lipids, but these components slowed the process of biodegradation [95], making the complex undesirable for clinical administration. To solve this biodegradation issue [112], redesign of the linker (e.g., ester, disulfide or phosphate bonds) and polar head group (e.g., guanidinium salt) were undertaken, introducing alternative hydrophobic chains or ionizable lipids (Fig. 5C).

Altogether, the effectiveness of a hydrophobic tail is determined by its number, length and degree of unsaturation. To enhance the transfection performance of cationic or ionizable lipids, hydrophobic tail should be designed to enhance the interaction between the cellular membrane and the mRNA-LNPs complex.

#### Heteroatom linker

The heteroatom linker acts as the bridge between the hydrophilic portion (polar head group) and the hydrophobic tail of a cationic or ionizable lipids (Fig. 5A). The linker plays an important role in determining the chemical stability, biodegradability, cytotoxicity, and transfection efficiency of the complex. According to its chemical structure, a heteroatom linker may be classified among several categories: ether, ester, amide, disulfide, acylhydrazone, arbamate, urea, phosphate bond, or other [113]. Of note, the design of the heteroatom linker must include consideration of its behavior in physiological pH and its potential as a target for enzyme actions [114, 115]. Some key advantages and disadvantages of different heteroatom linkers are briefly discussed below.

Multiple studies have shown that ether-bearing lipids promote more effective transfection than other degradable lipids incorporating ester or carbamate linkers [116, 117]. However, these cationic or ionizable lipids do not undergo normal degradation in vivo, indicating that the stable ether bond resists hydrolysis under physiological conditions and action of enzymes; this stability results in detectable cytotoxicity [118]. In contrast, ester linkages and carbamate-containing lipids can be cleaved by intracellular esterases. This feature allows the lipids to retain stability in circulation but reduces cytotoxicity compared to ether-bearing lipids [119]. Alternatively, amide-bearing cationic or ionizable lipids, such as dioctadecylamidoglycylspermine (DOGS; Fig. 5C), display reasonable stability and better transfection efficiency than ester or carbamate linkers due to their pH-buffering activities [120] and Coulombic repulsion [121]. In addition, some other heteroatom linkers have been recently introduced, including enzyme-cleavable linkers and photosensitive linkers. The initial reports indicate that these linkers can confer comparable transfection efficiencies but allow for controllable mRNA release due to the need for high localized enzyme concentrations [122, 123] or UV-induced cleavage to allow nucleic acid escape from endocytic vesicles [123]. In sum, the design of heteroatom linkers for cationic or ionizable lipids should include consideration of the number, spacing, orientation, and chemical structure of the linker group. These factors directly impact the chemical stability, biodegradability, transfection efficiency and cytotoxicity of the lipid that affect transfection characteristics in vitro and in vivo*.*

### Effectiveness of vaccines against different SARS-CoV-2 variants

Up to now, the WHO has authorized vaccines made by Pfizer-BioNTech, Moderna, AstraZeneca/Oxford, Janssen, Sinopharm, Sinovac, Bharat Biotech, and Novavax, as well as two vaccines from Serum Institute of India [20] (Table 1A). Most of these vaccines are administered according to a two-dose, prime/boost schedule with an interval of about 2–4 weeks. The viral vector-based vaccine from Janssen is an exception, as it only requires one dose. Comparing the protective efficacies of the SARS-CoV-2 vaccines in Phase III human trials, BNT162b2 [124], mRNA-1273 [125], and NVX-CoV2373 [61] had the highest, at 94.6%, 94.1%, and 89.7% respectively. Using pooled data from the UK and Brazil, AZD1222 [44] had a reported efficacy of 66.7%. Overall, the approved vaccines showed efficacies ranging from 50.4% to 94.6% [44, 61, 94, 124–129]; variations may be due to differences in clinical trial design, primary endpoint measurement, trial location, study population and prevalence of SARS-CoV-2 variants at the time of the trial. Though the reported efficacies of individual SARS-CoV-2 vaccines cannot be directly compared, analyzing the overall trends in efficacy data may help reveal how different vaccine platforms perform in terms of quality and/or quantity of immune response. Efforts toward developing vaccines against VOCs are summarized in Table 1B. This type of information could be crucial for determining which vaccine approaches are most suitable for future pandemics.

So far, five VOCs have been identified, each associated with a different wave of the COVID-19 pandemic; these include the Alpha, Beta, Gamma, Delta, and recent Omicron variants. Here, we describe key features of each VOCs and summarize current knowledge of how efficacious six selected WHO-approved vaccines are against the five VOCs.

### Alpha (B.1.1.7, UK) variant

Because the Alpha (B.1.1.7) variant was the earliest designated VOC, more data has been generated on vaccine effectiveness against this variant than subsequent VOCs. The Alpha variant has an N501Y mutation in the RBD of S protein, which enhances its affinity to the human ACE2 receptor [130] However, the mutations in Alpha variant only have a slight or no significant impact on vaccine efficacies. This conclusion was supported by several major clinical studies. For example, the NVX-CoV2373 vaccine has an 86% efficacy against the B.1.1.7 variant (compared to 96% efficacy against the original strain), according to a Phase III clinical trial conducted in the UK [131] (Table 2). The effectiveness of AZD1222 in preventing symptomatic nucleic acid amplification test (NAAT)-positive infection was 70.4% for B.1.1.7 and 81.5% for non-B.1.1.7 lineages [132].

**Table 2:** Protective efficacies of US FDA-approved COVID-19 vaccines toward five WHO variants of concern

SARS-CoV-2 Variants (WHO VOC)	Comirnaty (BNT162b2)	Spikevax (mRNA-1273)	Vaxzevria (AZD1222)	Janssen COVID-19 Vaccine (Ad26.COV2.S)	CoronaVac (COVID-19 Vaccine)	Nuvaxovid (NVX-CoV2373)
Alpha (B.1.1.7)	89.5% [233]	Reduced levels of neutralizing Abs [234]	70.4% [132]	70.1% (14 days after administration) 70.2% (28 days after administration) [235]	2.9-fold reduction in neutralizing Alpha variant [236]	86% [237]
Beta (B.1.351)	75% [233]	Reduced levels of neutralizing Abs [234]	N.D	38.1% (14 days after administration) 51.9% (28 days after administration) [235]	5.5-fold reduction in neutralizing Beta variant [236]	60% [237]
Gamma (P.1)	N.D	Reduced levels of neutralizing Abs [234]	N.D	36.4% (14 days after administration) 36.5% (28 days after administration) [235]	51%/4.3-fold reduction in neutralizing Gamma variant [236, 238]	N.D
Delta (B.1.617.2)	88%/90.9% Dose 2 (2-4W interval) [138, 239]	94.5% Dose 2 (2-4W interval) [138]	67%/82.8% Dose 2 (2-4W interval) [138, 239]	− 6% (14 days after administration) − 5.7% (28 days after administration) [235]	59%/3.4-fold reduction in neutralizing Delta variant [236, 240]	N.D
Omicron (B.1.1.529)	65.5% Dose 2 (2-4W interval) [138]	75.1% Dose 2 (2-4W interval) [138]	48.9% Dose 2 (2-4W interval) [138]	51.8% (14 days after administration) 51.9% (28 days after administration) [235]	12.5-fold reduction in neutralizing Omicron variant [236]	N.D

*W* Weeks, *N.D.* not determined or under investigation

### Beta (B.1.351, SA) variant and Gamma (P.1, Brazil) variant

In addition to the N501Y mutation, both B.1.351 (E484K and K417N) and P.1 (E484K and K417T) lineages carry mutations at K417 and E484. The N501Y, K417T and E484K mutations that were found to be associated with enhanced binding affinity to human ACE2 as well as increased transmissibility [133]. Both variants show a drastically loss of vaccine efficacy (Table 2).

### Delta (B.1.617.2, India) variant

The Delta variant carries L452R and T478K mutations within the S protein, which may stabilize the interaction between S protein and the ACE2 receptor of the host cell, thereby resulting in increased infectivity [134, 135]. A study in the UK showed that two doses of BNT162b2 or AZD1222 vaccines were respectively 88% and 67% effective against symptomatic disease caused by Delta variant. Moreover, comparing the two mRNA vaccines, mRNA-1273 (94.5%) conferred greater protection than BNT162b2 (90.9%) against the Delta variant (Table 2).

### Omicron (B.1.1.529, SA) variant

Among the five VOCs, the Omicron (B.1.1.529) variant is the latest to be designated as a VOC and has so far diverged into several descendent lineages, including BA.1, BA.2, BA.3 and the recently identified BA.4 and BA.5. Sequencing of early Omicron strains revealed more than 30 mutations in the S protein, which is more than the double number in the Delta variant (fewer than 15). These mutations result in enhanced transmission, higher risk of reinfection, and greater potential for humoral immune escape. The transmissibility of the Omicron variant is thought to be much higher than previous variants. This increased transmissibility may be due to the higher binding affinity of hACE2 to the Omicron RBD domain. Li et al. compared the hACE2 binding of several variants and found that BA.1.1 has higher binding affinity than other sub-variants. The reported binding order was as follows: BA.1.1 > BA.2 > BA.3≈BA.1 [136]. One case study even suggested that an asymptomatic individual infected with Omicron variant may have spread the virus across a corridor, suggesting an extraordinary level of hyper-transmissibility [137]. Results from a recently published study showed that vaccine effectiveness against symptomatic disease with the Omicron variant is notably lower than that against the Delta variant [138]. Vaccine effectiveness after two doses of AZD1222 vaccine against the Omicron variant was 48.9% at 2–4 weeks and dropped to almost no effect at 20 weeks after the second dose. In people who received two doses of mRNA vaccines, the effectiveness was higher (65.5% for the BNT162b2 and 75.1% for mRNA-1273 at 2–4 weeks after vaccination), but the efficacies fell to 8.8% and 14.9% by 25 weeks after the second dose [138]. Cele et al. compared the capacities of patient plasma to neutralize Omicron relative to the ancestral SARS-CoV-2 strain. Individuals who had received a third dose of BNT162b2 showed 25-fold higher antibody titers compared with those who had received two doses [139]. Chen et al. have reported that an individual who recovers from natural viral infection following two doses of mRNA vaccine can be expected to exhibit higher cross-variant neutralization capacity across different VOCs (including Omicron) than an individual who only received the two-dose mRNA vaccine regimen. This difference may be due to the fact that natural infection evokes a polyclonal antibody response to SARS-CoV-2 with a broader recognition range [140]. However, injection of a booster vaccine following two priming doses appears to be the most effective solution to combat the Omicron variant [141]. Due to the short follow-up time of studies to date, more work will be needed to understand the duration of vaccine effectiveness following a booster dose. Table 2 shows the summarized protective efficacy of US-FDA-approved COVID-19 vaccines towards above-mentioned VOCs.

According to news releases from Moderna on Jan 26, Mar 10, Jun 8, Jun 22, July 11, and Aug 15 of 2022, the company has manufactured an Omicron-specific booster vaccine (mRNA-1273.529) and started the Phase II study in January 2022. Another Phase II study was also initiated in March 2022 with Moderna’s Omicron-specific bivalent booster candidate (mRNA-1273.214), which combines mRNA-1273.529 and mRNA-1273. The results showed that mRNA-1273.214 exhibits an eightfold boost in neutralizing geometric mean titers (GMT) against Omicron among baseline-seronegative participants. Results of another Phase II/III study demonstrated that mRNA-1273.214 could induce a > fivefold boost in neutralizing antibodies against BA.4 and BA.5 subvariants. When administered to previously vaccinated and boosted participants, mRNA-1273.214 induced significantly higher neutralizing antibody response against BA. 4/5 as compared to mRNA-1273. The mRNA-1273.214 vaccine acquired conditional authorization by the Medicines and Healthcare Products Regulatory Agency in UK on August 15, 2022, which was the first approved Omicron vaccine in the world.

Every emergence of a new VOC presents a challenge to vaccine efficacy and has the potential to cause detrimental effects on human health. Therefore, the development of a pan-sarbecovirus vaccine would be ideal. Recently Liu et al. reported the development of a pan-sarbecovirus vaccine (CF501/RBD-Fc), which consists of an IgG Fc fragment-conjugated RBD of the SARS-CoV-2 WA1 strain as the immunogen and a novel small-molecule non-nucleotide STING agonist (CF501). Experiments in non-human primates (Rhesus macaques) showed that a booster dose of CF501/RBD-Fc increases the nAbs against most SARS-CoV-2 variants and may be expected to prevent infection caused by future VOCs [142, 143]. In addition, work by another research group highlighted the possibility of a providing a cross-clade booster of BNT162b2 mRNA vaccine in survivors of SARS-CoV-1 infection. Their findings suggested that it may be feasible to achieve pan-sarbecovirus neutralization via cross-clade boosting. The antibodies exhibit broad-spectrum potent activities and have been shown to neutralize not only SARS-CoV-2 VOCs but also sarbecoviruses identified in bats and pangolins with the potential to cause human infection [144]. Broadly-specific sarbecovirus vaccines have also been developed using a mosaic nanoparticle approach, which co-display RBDs from different clades of sarbecovirus phylogeny. Mosaic nanoparticle vaccines elicited broad neutralizing activity in mice and confer protection against heterotypic coronavirus challenges [145, 146]. Such attempts to develop a pan-sarbecovirus vaccine may someday yield a dream vaccine with potency against any future emerging VOCs or respiratory viruses.

### Effectiveness of vaccines in immunocompromised individuals

Immunocompromised individuals include blood cancer patients, organ transplant recipients, people with severe primary immunodeficiency, and patients who receive treatment with immunosuppressive medications. About 7 million people in the US and 0.5 million people in the UK are considered to be immunocompromised [147, 148]. Studies in Israel found that 40% of fully vaccinated hospitalized COVID-19 patients were immunocompromised due to corticosteroid therapy, chemotherapy, anti-CD20 treatment, or organ transplants [149]. Another study in the USA found that 44% of vaccine-breakthrough COVID-19 hospitalizations had immunosuppression [150]. Moreover, liver transplant recipients have a lower response to the BNT162b2 vaccine; only 47.5% of patients receiving the second BNT162b2 vaccine had a positive antibody response, and the average antibody levels were twofold less than those in healthy controls [151]. Similarly, only 37.5% of kidney transplant recipients with full BNT162b2 vaccination showed a positive response to S protein. In addition, the mean IgG anti-S protein level in seropositive kidney transplant recipients was significantly lower than that in controls [152]. Among patients who received immunosuppressive B cell-depleting therapy with rituximab (an anti-CD20 monoclonal antibody), only 58% had T cell-mediated immune responses after BNT162b2 vaccination, independent of their B cell-regulated humoral immune response [153].

According to a systematic review and meta-analysis, seroconversion rates after one vaccine dose were 16-fold less in organ transplant recipients than immunocompetent controls; seroconversion rates were also about half of control levels in patients with hematological cancers, immune-mediated inflammatory disorders, and solid cancers. A second vaccine dose significantly increased antibody responses across all patient groups, and a third dose conferred improved seroprotection in immunocompromised patients [154]. Other work further showed that a third dose of the BNT162b2 vaccine significantly improved the immunogenicity of immunocompromised patients, such as solid organ transplant recipients; 44% of patients who were seronegative after the first two doses of BNT162b2 vaccine became seropositive 4 weeks after the third dose [155]. In a cohort of 61 liver transplant recipients, Davidov et al. assessed anti-RBD IgG level, nAb titer and T cell levels before and after a third dose of BNT162b2 mRNA vaccine. The results of their study showed that humoral immune response increased from 56 to 98% after the third dose. The cellular response, anti-RBD IgG levels, nAb levels and T cell level also increase significantly after the third dose [156]. Moreover, kidney transplant recipients were given a fourth dose of the mRNA-1273 vaccine, and 66% of the patients displayed nAbs against the Delta strain. Without the fourth injection, only 16% of the patients displayed nAbs [157]. These studies have major implications for the utility of booster vaccines in patients with impaired immunity.

## Treatment of COVID-19

The treatments selected for COVID-19 patients depend on the severity of infection. Initially, exposure to the virus may cause mild symptoms that can be treated with medications such as paracetamol or ibuprofen [158]. For severe cases, WHO recommends the use of antiviral pills or intravenous infusion of therapeutic monoclonal antibodies. In this section, we highlight the antiviral drugs and therapeutic antibodies currently used for COVID-19 treatment.

### Small molecule antiviral agents

COVID-19 life cycle includes several steps to amplify the virus in the human body. Thus small molecule antiviral drugs are employed to interfere with virus life cycles such as impeding virus attachment with host cells, blocking proteolytic cleavage of S protein, and viral replication [159]. In this section, we discuss the action of EUAs approved small molecule antiviral drugs and the chemical structures are summarized in Additional file 1: Fig. S1.

#### Molnupiravir (Lagevrio®)

Perhaps the most promising orally administered small molecule treatment for COVID-19 is molnupiravir (EIDD-2801) [160]. This drug was initially discovered at Emory University and its biotechnology offshoot, DRIVE (Drug Innovation Ventures at Emory). Molnupiravir is a prodrug of EIDD-1931 (*N*^4^-hydroxycytidine, NHC), which was originally developed to treat Venezuelan equine encephalitis virus (VEEV) (EC_50_ = 0.43 μM).

EIDD-1931 is a ribonucleoside analogue, which resembles cytidine and potently inhibits influenza and other respiratory syncytial viruses. However, its utility is limited by poor oral bioavailability and rapid metabolism [161]. To address these issues, a prodrug, molnupiravir, was created to improve the pharmacokinetic profile. Molnupiravir is the 5′-isopropylester of EIDD-1931 and undergoes efficient hydrolysis to yield the parent drug after oral administration. After the hydrolysis step, EIDD-1931 is phosphorylated intracellularly to form EIDD-1931-triphosphate, which acts as a competitive substrate for RdRp of SARS-CoV-2. This action leads to an accumulation of errors and the inhibition of RNA replication.

Near the beginning of the COVID-19 pandemic, EIDD-1931 was tested and showed high potency against SARS-CoV-2 (EC_50_ = 3.4 μM and EC_90_ = 5.4 μM) [162]. To determine whether molnupiravir might be an orally efficacious for SARS-CoV-2 treatment, the therapeutic efficacy of the prodrug was evaluated in a ferret model, where it significantly reduced the virus titer within 12 h after dosing. On March 23, 2020, DRIVE (not-for-profit biotechnology company) and Ridgeback Biotherapeutics announced a licensing deal in which Ridgeback Biotherapeutics gained exclusively license to DRIVE's EIDD-2801 for conducting the necessary trials against COVID-19. On May 26, 2020, Merck and Ridgeback Biotherapeutics entered into a collaboration agreement to develop molnupiravir for the treatment of patients with COVID-19. The drug was further evaluated with various dosing regimens in the Phase II trial (NCT04405570), and the results showed that a regimen of 800 mg, twice daily for five days was able to proceed to the next stage. The Phase II/III clinical trials on molnupiravir were started in 2021. The MOVe-IN (NCT04575584) and MOVe-OUT (NCT04575597) studies respectively targeted hospitalized and non-hospitalized patients [163]. The promising results from clinical trial convinced the FDA to issue an EUA for use of molnupiravir as a treatment for adults with mild to moderate COVID-19 illness. On Dec 23, 2021, the drug was released with the brand name Lagevrio®, based on 30% reduction in hospitalizations and deaths from MOVe-OUT trial. Currently, the Phase III clinical trials of molnupiravir are still ongoing.

#### Nirmatrelvir (Paxlovid®)

Apart from RdRp inhibition by ribonucleoside analogues, 3C-like protease (3CL^pro^) is another important drug target for anti-SARS-CoV-2. Nirmatrelvir (PF-07321332) is an antiviral agent developed by Pfizer that is administrated with ritonavir (an inhibitor of cytochrome P450 3A4) for the treatment of mild-to-moderate COVID-19 in adults and people 12 years of age and older.

Scientists from Pfizer started the development of this treatment by screening their in-house compounds, and they identified an intravenously administered candidate, lufotrelvir (PF-07304814), which had been originally developed to target SARS-CoV-1 in 2003. This potential antiviral agent was then tested in a Phase I clinical trial to explore its safety and efficacy (NCT04627532 and NCT04535167) in 2020. However, the peptide-like nature of lufotrelvir necessitates intravenous administration, which may severely limit its utility for non-hospitalized patients. Therefore, an effort to apply peptidomimetics for optimization of the drug was undertaken by Dr. Dafydd Owen and his team at Pfizer Medicinal Chemistry. This effort yielded nirmatrelvir (PF-07321332) in mid-2020, which exhibits reduced numbers of hydrogen bond donors and free rotatable bonds. In addition, nirmatrelvir has a rigid bicyclic non-canonical amino acid (fused cyclopropyl ring with two methyl groups), which mimics the leucine residue. This feature was inspired by the key component of an HCV NS3/4A inhibitor (boceprevir), and helped to improve the pharmacokinetic profile of nirmatrelvir, increasing the oral bioavailability from 1.4% (for PF-00835231) to 50%.

In the development stage, nirmatrelvir was combined with ritonavir. The ritonavir inhibits cytochrome P450 activity to slow the metabolism of nirmatrelvir (metabolized by P450 3A4). This approach has been previously applied for HIV treatment. The results of a Phase I clinical trial revealed that the nirmatrelvir and ritonavir combination was safe and well tolerated. Moreover, the Phase III trial (NCT04960202) showed that nirmatrelvir/ritonavir decreased the risk of progression to severe COVID-19 by 89%. On December 22, 2021, the US FDA issued an EUA for nirmatrelvir/ritonavir (Paxlovid®), making it the first orally administered direct antiviral drug to be approved for SARS-CoV-2 treatment. Currently, several Phase III clinical trials of nirmatrelvir/ritonavir are ongoing in the US and Asia [164].

As announced in a news release on Jan 22, 2022, the WHO recommends nirmatrelvir and ritonavir as a highly effective COVID-19 therapy, but a lack of price transparency and high costs have limited their supply in developing countries.

#### Ensitrelvir (S-217622, Xocova®)

On July 26, 2021, the Japanese pharmaceutical company, Shionogi, announced its COVID-19 therapeutic agent ensitrelvir, an orally administered 3C-like protease inhibitor. The Phase I clinical trial was initiated in Japan. According to the latest data from the Phase II/III clinical trial, ensitrelvir elicits rapid reductions in viral titer and viral RNA, and up to now, no serious adverse events have been observed. Thus, ensitrelvir has promise to be a highly efficacious and safe oral drug for use against COVID-19 [165].

In the development of this drug, researchers at Shionogi targeted 3CL protease to influence viral replication, as protease inhibitors have been successfully used as treatments for HIV and hepatitis C virus. However, most 3CL protease inhibitors are peptide-like compounds, which have poor stability in vivo, low membrane permeability, and undesirable pharmacokinetic profiles. Consequently, the research team from Shionogi aimed to identify small-molecule SARS-CoV-2 3CL protease inhibitors.

The hit identification stage was performed by applying structure-based drug design based on identified interactions between known inhibitors and the binding site of 3CL protease. The scientists optimized the best hit compound according to interactions mapped from co-crystallization with 3CL protease. This optimization process led to identification of a lead compound that exhibited 90-fold greater potency in the enzymatic assay than the initial hit compound and a reasonable pharmacokinetic profile. Further compounds were then designed and synthesized from this new lead compound, resulting in the discovery of ensitrelvir (S-217622). Ensitrelvir showed potent biochemical activity: IC_50_ value of 0.013 μM in the enzymatic assay and an EC_50_ value of 0.37 μM. Furthermore, it had superior drug metabolism and pharmacokinetic profiles, including excellent oral absorption in rats, dogs, and monkeys. Moreover, ensitrelvir was shown to act as a broad-spectrum antiviral against currently identified variants of coronaviruses, and it is a safe oral drug without any observed toxicity. The antiviral efficacy of ensitrelvir was examined in mice infected with SARS-CoV-2 Gamma strain. Ensitrelvir was dosed 12 h after infection, and the viral titers were evaluated after another 24 h. In the mice, ensitrelvir not only significantly and rapidly reduced the viral activities and loads, but also showed a desirable preclinical profile. On this basis, ensitrelvir was advanced to further evaluation in clinical trials.

In a Phase I clinical trial to test tolerability and safety, there were no major clinical adverse events identified. Unlike paxlovid, ensitrelvir does not require repeated dosing to achieve efficacious levels. In the Phase II/III clinical trial, the efficacy and safety of orally administered ensitrelvir were evaluated; patients with mild COVID-19 or asymptomatic SARS-CoV-2 infection were dosed once daily for five days. During the trial, the positive viral titers of patients decreased by approximately 60–80% within the five days, and there were no exacerbation cases which necessitated hospitalization in the ensitrelvir group. Currently, this joint research effort between Hokkaido University and Shionogi has progressed to global Phase III trials.

#### Remdesivir (Veklury®, GS-5734)

Remdesivir is a broad-spectrum intravenously administered antiviral drug originally developed by Gilead Sciences in 2009 to treat hepatitis C and respiratory syncytial virus (RSV). The drug was subsequently investigated for use against Ebola virus disease and Marburg virus infections, as well as Coronaviridae family viruses exemplified by MERS and SARS [166]. Remdesivir is a monophosphoramidate prodrug of an adenosine analog GS-441524 [167]. After biotransformation, GS-441524 triphosphate acts as a ribonucleotide analogue inhibitor of viral RdRp [168].

Remdesivir is an adenosine analogue, which is incorporated into nascent viral RNA chains and causes pre-mature termination. Remdesivir possesses a 10-substituted 4-aza-7,9-dideazaadenosine C-nucleoside, which improves its chemical stability and resistance to enzymatic deglycosylation reactions. The designers of this drug also cleverly introduced a 1′-CN modification, which sterically clashes with RdRp (residue S861) upon chain elongation, providing selectivity for viral polymerases and preventing significant toxicity. During the COVID-19 pandemic, remdesivir was quickly repurposed based largely on in vitro cell-based assays against SARS-CoV-2 and related coronaviruses. These assays demonstrated an IC_50_ of 770 nM and an IC_90_ equal to 1,760 nM (with cytotoxic concentration > 100 μM, SI > 129.87) [167].

On October 22, 2020, remdesivir became the first treatment for COVID-19 to receive FDA approval. The approval was based primarily on three clinical trials (NCT04280705, NCT04292899, and NCT04292730) of 2,043 hospitalized participants with COVID-19 treated under an EUA originally issued on May 1, 2020. In light of the Omicron variant surge, the FDA expanded the indication for remdesivir to include treatment of outpatients with mild-to-moderate COVID-19 [169, 170].

The antiviral agents currently in clinical trials for use against COVID-19 are summarized in Additional file 1: Table S2.

### Therapeutic antibodies

Due to their high specificity and versatility, monoclonal antibodies have become crucial tools for the treatment and diagnosis of various diseases, including virus infections [171]. As of March 2022, more than 100 monoclonal antibodies have been approved by US FDA for use as drugs, and new approvals continue to accumulate [172, 173]. Traditionally, therapeutic antibodies have been generated by mouse hybridoma techniques coupled with antibody humanization protocols. However, the use of mouse-derived antibodies carries a risk of immunogenic response to murine sequences, so fully human antibodies have been increasingly used as therapeutic products over the last few years. Three main platforms are utilized to generate fully human antibodies, including phage display, transgenic mice, and single B cell isolation. The major primary indications for therapeutic antibodies are cancer (45%) and immune-mediated disease (27%) [172]. nAbs have also been developed for use against infectious diseases. These drugs are often able to reduce disease progression immediately after administration, regardless of whether the patient has fully developed immunity [174, 175]. For example, a humanized Ab, palivizumab, was approved for use against RSV in 1998, as it can provide immuno-prophylaxis for pediatric lower respiratory tract infections [176]. Furthermore, the Ebola virus S glycoprotein-binding monoclonal antibodies, REGN-EB3 and ansuvimab (MAb114), were respectively made from VelocImmune mice and B cells of Ebola convalescent patients. Both of these treatments have successfully improved patient outcomes, reducing the overall mortality of Ebola to ~ 35% in all patients [177]. In the fourth quarter of 2020, the US FDA approved REGN-EB3 (Inmazeb) and ansuvimab for the treatment of Zaire ebolavirus infection [178].

Collaborative efforts of governments and biopharmaceutical industries have facilitated the rapid authorization of vaccines against COVID‑19. Nevertheless, the coronavirus pandemic remains a serious global concern. About 2% of the global population is thought to be at increased risk for insufficient response to COVID-19 vaccines [179], and recent evidence suggests that protecting vulnerable populations from SARS-CoV-2 infection could help prevent evolution of the virus, which is a key factor in the emergence of variants [180]. Therefore, administering nAbs with high prophylactic potency can serve to protect these vulnerable populations and reduce the probability of viral evolution.

A number of monoclonal antibodies have been applied to treat and detect COVID-19. As of May 2022, over 35 nAbs have been studied in clinical trials [181]. Numerous countries have authorized the emergency use of anti-SARS-CoV-2 nAbs, and full approvals have also been granted in a few selected cases. The fully approved nAbs are limited to Regkirona (regdanvimab) in South Korea and EU, as well as Xevudy (sotrovimab) and REGEN-COV in EU and UK. In the following section we focus our discussion on eight nAbs that have received EUA from the US FDA, including bamlanivimab, etesevimab, REGEN-COV (casirivimab and imdevimab), Xevudy (sotrovimab), Evusheld (cilgavimab and tixagevimab), and bebtelovimab. Moreover, the Omicron variant became the dominant strain within two months of its emergence in November 2021 [182]. It carries 15 mutations in the RBD of S protein, which severely impact the neutralizing activity of available nAbs. Therefore, we also discuss the mechanisms underlying resistance of the Omicron variant to nAb drugs.

#### EUA for COVID-19 therapeutic mAbs

##### REGEN-COV (Casirivimab and Imdevimab)

The antibody cocktail of casirivimab and imdevimab was developed by Regeneron pharmaceuticals to target the RBD of SARS-CoV-2. Casirivimab is a humanized Ab generated from VelocImmune transgenic mice immunized with a plasmid expressing SARS-CoV-2 S protein [174]. Imdevimab was isolated from single B cells of convalescent patients with SARS-CoV-2 infection [13]. In November 2020, the US FDA issued an EUA for the intravenous infusion of combined casirivimab and imdevimab for the treatment of mild to moderate COVID-19 treatment in adults and pediatric patients over 12 years of age who test positive for SARS-CoV-2 infection and have high risk of progression to severe COVID-19, or elderly patients with chronic disease [183]. Although these antibodies do not have modifications in the Fc region, they still initiate antibody-mediated cytotoxicity and cellular phagocytosis according to i*n vitro* assays [184]. Treatment of casirivimab and imdevimab was shown to prevent escape mutations in S protein of SARS-CoV-2 and displayed therapeutic effects toward several SARS-CoV-2 variants, such as Alpha, Beta, Gamma and Delta variants [174, 175, 185]. However, these broadly protective SARS-CoV-2 nAbs appear to be ineffective against the Omicron variants. [185–188]. The residual BA.2-neutralizing activity of imdevimab was even lower when tested against BA.4/BA.5. Meanwhile, casirivimab’s neutralizing activity was absent for all tested Omicron variants, including BA.2 and BA.4/BA.5 [189]. Due to the Omicron BA.2 variants escaping from REGEN-COV neutralization, the US FDA paused the use of this combination treatment for COVID-19 since January 24, 2022 [187, 190] (Table 3).

**Table 3:** EUA (US FDA) and approved anti-SARS-CoV-2 therapeutic antibodies

Antibody	US EUA date	Approved	Pause to use	Manufacturer	References
Bamlanivimab	11/09/2020	–	U.S. (04/16/2021)	Eli Lily and Company	[11, 12]
REGEN-COV (Casirivimab + Imdevimab)	11/21/2020	EU, UK, Japan	U.S. (01/24/2022)	Regeneron pharmaceuticals	[13–16]
Bamlanivimab + Etesevimab	02/09/2021	–	U.S. (01/24/2022)	Eli Lily and Company	[11, 241]
Xevudy (Sotrovimab)	05/26/2021	EU, UK	U.S. (03/30/2022)	GlaxoSmithKline plc and Vir Biotechnology, Inc	[196]
Evusheld (Cilgavimab + Tixagevimab)	12/02/2021	EU, UK	–	AstraZeneca	[204]
Bebtelovimab	02/11/2022	–	–	Eli Lily and Company	[242]
Regkirona (Regdanvimab)	–	EU, South Korea	–	Celltrion HealthCare	[243]

*EU* European Union, *EUA* Emergency Use Authorization

##### Bamlanivimab and Etesevimab

In September 2021, the US FDA granted an EUA for the therapeutic use of combined bamlanivimab and etesevimab, which was developed by Eli Lily and Company. The administration of bamlanivimab and etesevimab by intravenous infusion was approved for use in adult and pediatric patients over 12 years old as well as elderly patients with mild to moderate COVID-19 symptoms [191]. Both bamlanivimab and etesevimab were generated by isolating antigen-specific B cells from patients convalescing from COVID-19; the two nAbs target different but overlapping epitopes within the RBD of S protein of SARS-CoV-2 [11, 12, 175]. Etesevimab contains LALA substitutions at residues 234 and 235, which nullifies Fc-mediated effector functions (Table 3) [175]. Although bamlanivimab monoclonal antibody was granted an early EUA in November 2020 [192], it failed to target the SARS-CoV-2 variants with mutations at residues 484 and 493 in the RBM; this lack of effect weakens the protection efficacy to Beta, Gamma, as well as the all Omicron variants including BA.1, BA.2 and BA.4/BA.5 sublineages [174, 185, 187, 189]. Treatment with bamlanivimab alone was also ineffective for the Delta (B.1.617.2) variant, but its combination with etesevimab could partially neutralize the Delta variant [174, 186, 193]. The Omicron sublineage, including BA.4/BA.5, has escaped the neutralizing activity of etesevimab [189]. Based on these reports, the combination of bamlanivimab and etesevimab is effective at neutralizing Alpha and Delta, but not others, including Omicron BA.2 variants that leading the US FDA restricted the usage of bamlanivimab and etesevimab since January 24, 2022 (Table 3) [185–187, 190, 194, 195].

##### Xevudy (Sotrovimab)

Sotrovimab was developed by GlaxoSmithKline and Vir Biotechnology, Inc. This nAb was isolated from memory B cells of a patient with SARS-CoV-1 infection [196]. Sotrovimab was granted an EUA by the US FDA in May 2021 to treat adult and pediatric patients over 12 years of age with mild-to-moderate COVID-19 [197]. Engineering of sotrovimab was undertaken to enhance the activation of Fc-effector functions, including antibody-dependent cell cytotoxicity and antibody-dependent cellular phagocytosis, resulting in immune-mediated viral clearance [196]. This nAb also exhibits enhanced engagement with the neonatal Fc receptor (FcRn), and the antibody half-life was extended by substituting the LS amino acid residues (M428L/N434S) in the Fc region (Table 4) [198]. The mAb does not block the interaction between viral S protein and host ACE2 receptor, but it instead targets RBD epitopes that are shared across sarbecoviruses, allowing it to neutralize of a variety of VOCs, including Beta, Gamma, Delta and Omicron BA.1 [199]. However, sotrovimab exhibits poor neutralization of Omicron variants, including BA.2 and BA.4/BA.5. [25, 187, 199–201]. Due to resistance of BA.2, the US FDA announced that sotrovimab is no longer authorized for treatment of COVID-19 (Table 3) [202].

**Table 4:** Summary of FDA EUA engineering mAbs

Antibody	Fc Variants	Binding Affinity				Effector Function	USPTO application	Status of patent
		**FcγRI**	**FcγRIIa**	**FcγRIIIa**	**FcRn**			
Xevudy (Sotrovimab)	M428L/ N434S	N.D	** + ** [196]	** + ** [196]	** + ** [205]	•Enhanced ADCC •Enhanced ADCP •Enhanced IgG half life	11/124,620	Patented
Evusheld (Cilgavimab + Tixagevimab)	L234F/ L235E/ P331S	** − ** [244]	** − ** [205]	N.D	N.D	•Reduced ADCC •Reduced CDC •Enhanced IgG half life	16/159,451	Patented
	M252Y/ S254T/ T256E	** − / + ** [245]	** − ** [245]	** − ** [245]	** + ** [205, 246]		13/133,845	Patented
Etesevimab	L234A/ L235A	** − ** [205, 247]	** − ** [247]	N.D	** + ** [205]	•Reduced ADCC •Reduced CDC •Enhanced IgG half life	15/210,464	Abandoned

Binding affinity of Fc engineered antibodies were compared to wild type antibodies of IgG. Abbreviations: ADCP, antibody-dependent cellular phagocytosis; ADCC, antibody-dependent cell cytotoxicity; CDC, complement-dependent cytotoxicity. -/ + : no change; -: reduction; + : enhancement; ND: no data

##### Evusheld (Cilgavimab and Tixagevimab)

The cocktail of cilgavimab and tixagevimab (called Evusheld) was developed by AstraZeneca for the prevention of COVID-19 infection. This treatment is administered by intramuscular (IM) injection to individuals over 12 years old who are unable to be vaccinated against COVID-19 due to severe allergy history or who are immunocompromised [203]. In December 2021, the US FDA first authorized the antibody combination for pre-exposure prevention of COVID-19 for up to 12 months [203]. Cilgavimab and tixagevimab recognize non-overlapping sites of the RBD and block the interaction between virus to host ACE2 receptor [204]. These mAbs have modified amino acid residues in the Fc region that reduce the potential risk of effector functions as well as complement binding (i.e., L234F/L235E/P331S substitutions) [205]. In addition, the inclusion of an optimized Fc region with M252Y/S254T/T256E substitutions extended the half-life of the antibodies by enhancing antibody binding to human FcRn [205, 206]. Using an in vitro live-virus focus reduction neutralization assay (FRNT), the cocktail mAbs were shown to inhibit SARS-CoV-2 variant, including Alpha, Beta, Gamma, and Omicron (including BA.1 and BA.2), although the neutralizing activity was lower for Omicron compared with the other VOCs [185, 187]. Another study indicated the cocktail of mAbs still retains activity against Omicron variants BA.4/BA.5, although this activity is eightfold reduced as compared with BA.2 [189, 201]. Recently, the US FDA increased the initial dosage from 150 mg of cilgavimab and 150 mg of tixagevimab to 300 mg each of cilgavimab and tixagevimab with repeated dosages every six months [207].

##### Bebtelovimab

In February 2022, the US FDA issued an EUA for Eli Lily’s monoclonal antibody, bebtelovimab, to treat the mild to moderate COVID-19 adult and pediatric patients over 12 years of age [208]. Bebtelovimab targets RBD and was generated from a single B cell isolated from a convalescent patient with COVID-19. It was shown to effectively neutralize several authentic SARS-CoV-2 VOCs, the IC_50_ values ranged from 4 to 16 ng/ml [209]. In addition, bebtelovimab retains activity toward VOCs with RBD mutations of K417N, L452R, E484K and N501Y. This conclusion was supported by pseudovirus neutralization assays showing that it has potent neutralizing activity against Alpha, Beta, Gamma, Delta, Omicron variants including BA.1, BA.2 as well as BA.4/BA.5 [201, 209].

#### Neutralizing antibodies for Omicron variants

**Fig. 6:**
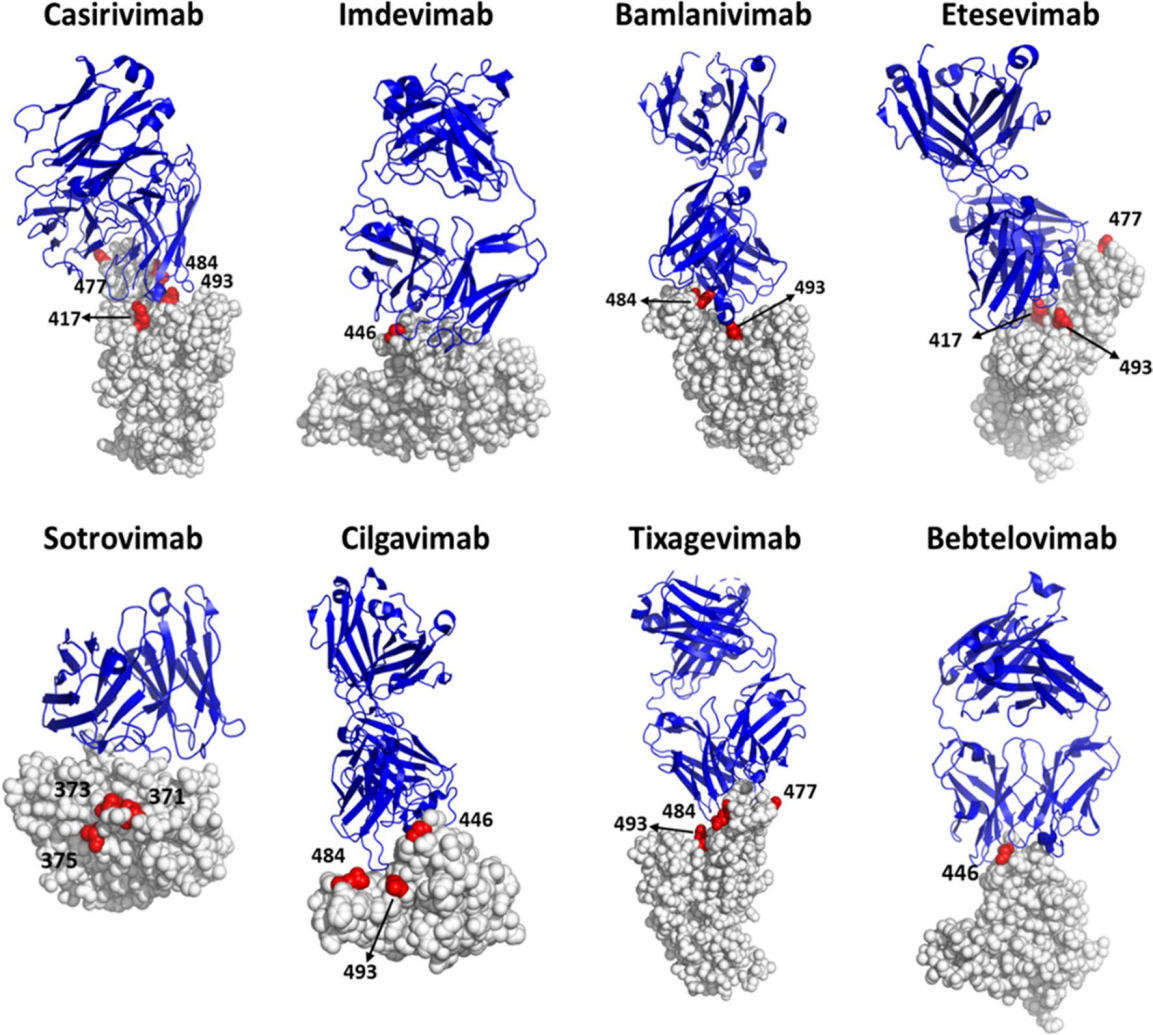
Structure of nAbs binding to RBD. The potent escape mutations in BA.1 variant were indicated in red. The Fab region of antibody show in Blue ribbon and RBD represent as white spheres. Complexes are visualized with PyMOL Molecular Graphics System, v2.5.2 (Schrödinger, LLC) software. The protein data bank (PDB) accession codes for the structures shown are 6XDG (casirivimab and imdevimab), 7KMG (bamlanivimab), 7C01 (etesevimab), 7R6W (sotrovimab), 7L7E (cilgavimab and tixagevimab), and 7MMO (bebtelovimab)

The Omicron variants have been shown to evade most SARS-CoV-2 nAbs. The original Omicron (B.1.1.529) variant carries 15 mutations in the S protein RBD, including G339D, S371L, S373P, S375F, K417N, N440K, G446S, S477N, T478K, E484A, Q493R, G496S, Q498R, N501Y, and Y505H. These mutations have led to greatly reduced neutralization potencies of etesevimab, bamlanivimab, REGEN-COV (casirivimab and imdevimab), Evusheld (cilgavimab and tixagevimab), bebtelovimab, and Xevudy (sotrovimab) [210]. The structure of nAbs binding to RBD is shown in Fig. 6, and the red dots indicate mutation sites [185, 211]

The epitopes of etesevimab [212] overlap with the ACE2 binding site and may be modified by RBD mutations at K417, S477 and Q493 [211]. In particular, K417N disrupts a critical salt bridge interaction between K417 and a negatively charged residue in the antibody [210]. The nAbs such as tixagevimab, and casirivimab are very sensitive to changes in K417, S477, E484, T478 and Q493. Tixagevimab significantly reduced binding affinity to the Omicron variant RBD, potentially due to the S477N, T478K, and Q493R mutations whereas K417N, E484A, S477N, and Q493R would lead to steric clashes with casirivimab.

Bamlanivimab bind to the right shoulder of the RBD [12]. The activities of this nAbs is highly susceptible to changes at E484 and Q493. Thus, the E484A, and Q493R mutations in Omicron attenuates neutralization by these antibodies. Other antibodies that bind the right shoulder of the RBD include imdevimab [13] and cilgavimab. These two nAbs bind further down the RBD right shoulder, toward the S309 site. Moreover, a loop formed by RBD residues 440–449 is critical for the binding of imdevimab and cilgavimab [213], rendering these antibodies sensitive to changes at N440, K444, G446 and N448. Therefore, the G446S mutation substantially affects the abilities of these antibodies to neutralize Omicron. Even if similar nAbs could tolerate a G446S single mutation, the N440K/G446S or E484A/Q493R combination may significantly reduce their binding affinity [210, 211]. Thus, Omicron was shown to escape imdevimab and cilgavimab.

Sotrovimab retains strong RBD binding capability. This binding is rather unexpected, as G339 and N440 are part of the epitopes, and Omicron carries G339D and N440K mutation [211]. However, the IC_50_ of sotrovimab is reduced to 181 ng/mL and may be subject to further reduction against Omicron sub-variants with R346K mutations [210]. Sotrovimab nAb targets a cryptic site in the RBD that is generally not exposed, making its neutralizing activity weaker than that seen for other nAbs [213]. This antibody is sensitive to changes at F374, T376 and K378, and a loop involving RBD residues 371–375 lies in the ridge [210].

Bebtelovimab retains neutralization ability against Omicron BA.1 and BA.2. The binding epitopes of bebtelovimab has been identified as K444, V445, G446, and P499, which are relatively conserved epitopes of all known variants [209]. Although structure basis study found that G446S potential clash the binding ability to RBD. However, S446 loop has the flexibility to allow bebtelovimab binding, resulting in a slight impact on their interaction [185]. In addition, both N439 and N501 are part of epitopes of bebtelovimab. However, the mutation on these amino acids did not affect its function [185, 209].

Currently, Omicron variants are the predominant circulating strains around the world, and these variants can evade many therapeutic antibodies. To address this problem, Lu et al. recently used the mRNA-lipid nanoparticle immunization method to generate a set of Omicron-targeting monoclonal antibodies. Five of these K-RBD-mAbs showed strong binding and neutralizing activities toward all SARS-CoV-2 variants of concern. Chimeric derivatives of these five antibodies could also neutralize Omicron sublineages BA.1 and BA.2 with low IC_50_ values (ranging from 5.7 to 12.9 ng/mL) [90]. In another study, the researcher screened the SARS-CoV-2 antibodies from mice immunized with the viral spike protein. Among these screened antibodies they found that the most potent monoclonal antibody was named SW186. Peculiarly, SW186 showed the best neutralizing activity against SARS-CoV-2 VOCs and SARS-CoV-1 also. SW186 reduced the viral loads in the mice lungs after infection with SARS-CoV-2 VOCs. A distinct feature of SW186 is the binding epitope, Cryo-EM structure showed that this antibody was bound to an outer surface of RBD, which was distinct from RBM that bound to ACE2 [214]. Thus, these antibodies can potentially be developed as universal nAbs against SARS-CoV-2. Table 5 summarizes the activities of nAbs against SARS-CoV-2 Omicron variant.

**Table 5:** Neutralization of the Omicron variant BA.1 by the EUA-granted therapeutic Abs

		IC_50_ (ng/mL) for Omicron BA.1								
Antibody	Epitope	Peking U. [210]	Vir Biotech [199]	Columbia U. [248]	Göttingen U. [249]	Washington U. [188]	Oxford U. [249]	Université de Paris [250]	Tokyo U. [187]	The U.S. NIH [185]
Casirivimab	RBM	> 10,000	> 10,000	> 10,000	> 1,000	> 10,000	> 10,000	> 9,000	14,110	> 10,000
Imdevimab	RBD	> 10,000	> 10,000	> 10,000	> 10,000	> 10,000	> 10,000	> 9,000	> 50,000	> 10,000
REGEN-COV (Casirivimab + Imdevimab)		N.D	> 10,000	> 10,000	> 1,000	> 10,000	N.D	> 9,000	> 10,000	N.D
Bamlanivimab	RBD	> 10,000	> 10,000	> 10,000	> 10,000	> 10,000	> 10,000	> 9,000	> 50,000	> 10,000
Etesevimab	RBM	> 10,000	> 10,000	> 10,000	> 10,000	> 10,000	> 10,000	> 9,000	> 50,000	> 10,000
Bamlanivimab + Etesevimab		N.D	> 10,000	> 10,000	> 10,000	> 10,000	N.D	> 9,000	> 10,000	N.D
Xevudy Sotrovimab	RBD	181	~ 200	100 ~ 1,000	100 ~ 1,000	373	256	917	373	281
Cilgavimab	RBM	2,178	2,772	~ 1,000	N.D	381	3,488	1,213	443	5850
Tixagevimab	RBM	6,860	> 10,000	100 ~ 1,000	N.D	913	1,152	8,305	1,299	269
Evusheld (Cilgavimab + Tixagevimab)		N.D	418	10 ~ 100	N.D	147	273	773	225	N.D
Regdanvimab	RBM	N.D	> 10,000	N.D	N.D	> 10,000	N.D	> 9,000	N.D	> 10,000
Bebtelovimab	RBD	N.D	N.D	N.D	N.D	N.D	N.D	N.D	N.D	5.1
	Approach	Pseudovirus	Pseudovirus	Pseudovirus	Pseudovirus	Authentic virus	Authentic virus	Authentic virus	Authentic virus	Pseudovirus

*N.D.* not determined

#### Market for COVID-19 therapeutic mAbs

In January 2021, the US government signed a contract to purchase 1.25 million doses of REGEN-COV and paid $2.625 billion to Regeneron ($2,100/dose). In January 2021, the German government purchased 200,000 doses at a price of $488 million ($2,440/dose). In total, Regeneron received more than $6.19 billion for the production of therapeutic Abs (REGEN-COV) against COVID-19 in 2021 (Regeneron 2021 Full Year Financial Reports).

Bamlanivimab was the first SARS-CoV-2 nAb to receive an EUA from the US FDA for clinical use. The drug was supplied by Eli Lily to the US government in 300,000 vials of 700 mg doses for $375 million ($1,250/dose) [174]. Due to mutations at E484 in the S protein, the Beta, Gamma, and Delta variants were completely refractory to bamlanivimab neutralization. The US FDA therefore revoked the EUA of bamlanivimab monotherapy for COVID-19 patients on April 9, 2021. According to Eli Lily's 2021 financial report, revenue from COVID-19 antibodies, which include bamlanivimab alone as well as bamlanivimab combined with etesevimab, was $871 million in 2020 and $2.24 billion in 2021. It is worth noting that bamlanivimab and etesevimab administered together were not effective against several variants, including Gamma, Beta, Delta and Omicron. In January 2022, the FDA revised the EUA to limit the use of these drugs to situations in which the patient is likely to have been infected with or exposed to a variant that is susceptible to this combination treatment.

Sotrovimab, was granted an EUA by the US FDA in May 2021. In January 2022, GSK and Vir Biotechnology secured binding agreements for the sale of approximately 1.7 million doses of sotrovimab worldwide and provided 0.6 million doses to the US government in Q1 2022. AstraZeneca has signed an agreement with the US government to supply 1.7 million doses of the long-acting antibody combination Evusheld (tixagevimab and cilgavimab) for the prevention of COVID-19; the total value of the agreement for Evusheld is $855 million ($503/dose). Eli Lily's bebtelovimab can neutralize Omicron, including the sub-variant BA.2, as demonstrated by assays with pseudovirus and authentic virus [185, 209]. The company announced an agreement with the US government to supply up to 600,000 doses of bebtelovimab for at least $720 million ($1,200/dose) no later than March 31, 2022. The US government will then have an option to buy 500,000 more antibody doses before July 31, 2022.

nAb drugs have made great contributions to combatting COVID-19 over the past two years. However, several factors contribute to concerns about the future demand for nAbs. For example, superior or competing therapies have emerged, such as easier-to-use therapeutics like oral antiviral drugs. In addition, it is more common for individuals to have experienced some preventive circumstance, such as vaccination or stimulation of natural immunity after infection by less dangerous variant such as Omicron. Furthermore, the unpredictability of virus mutations adds uncertainty to the value of nAb development efforts. Based on the emergence of new variants, the FDA has revised and may further revise EUAs for COVID-19 antibodies according to the degrees of efficacy against the most prevalent variants. Eli Lily and Regeneron have forecasted limited revenue from the sale of antibodies after the first quarter of 2022. Even so, past clinical evidence suggests that therapeutic antibodies can still make a substantial contribution to the treatment of COVID-19, and these agents may help to resolve this pandemic in the near future. Scientists continue to work hard studying novel and broadly neutralizing antibodies, and these efforts may yet pay off [90]. The fact that bebtelovimab recently obtained an EUA from the US FDA due to its high neutralizing effectiveness against Omicron BA.1 and BA.2 is a good example of the current potential for nAbs [185].

## Conclusions and future perspectives

Since the first outbreak of SARS-CoV-2 infections in late 2019, several major VOCs have emerged, including Alpha, Beta, Gamma, and Delta. Most recently, the Omicron variant which was initially identified on November 24, 2021 in South Africa and Botswana, has rapidly spread throughout many countries and quickly replaced Delta as the dominant variant circulating in the world. Within a very short time, Omicron was detected globally [137, 139]. Since the spring of 2022, BA.4 and BA.5 have been detected throughout the world. Daily reports of variant tracking show that BA.5 has spread faster than BA.2 and became the dominant variant of SARS-CoV-2 in Asia, Europe and Oceania by the middle of June 2022. Currently, BA.5 is the predominant subvariant globally. Compared to the original virus, Omicron contains over than 30 mutations in S protein, including 15 mutations in the RBD, and seven mutations in the NTD, and three mutations near the furin cleavage site. These mutations are known to confer resistance to neutralization by antibody drugs, sera of convalescent patients and vaccinated individuals [188, 215]. Therefore, an urgent need remains to generate new tools to combat Omicron and future SARS-CoV-2 VOCs.

mRNA-based technologies have been perhaps the most successful platform for rapidly developing vaccines against SARS-CoV-2, as evidenced by the widespread use of the BNT162b2 of Pfizer-BioNTech and mRNA-1273 by Moderna, as well as the manufacturing of mRNA-1273.214 of Moderna, which is possibly the first Omicron-specific booster to be authorized probably in Fall of 2022. Advantages of mRNA vaccines include: 1) a cell-free, safe, and time-saving manufacturing process that does not require large-scale growth of highly pathogenic organisms and has reduced risk of contamination by dangerous pathogens, 2) no need for a dedicated product-specific production facility, and 3) only requires alteration of the RNA sequence to change to the target protein, leaving the physiochemical characteristics of the drug product largely unaffected. These advantages allow for a streamlined manufacturing process that is amenable to accelerated, cost-effective mRNA vaccine development and mass production [216].

Current clinical trial data suggest that the approved mRNA-based COVID-19 vaccines are safe and effective for most of the population [125, 217]. However, there are still rare cases of severe local and systemic reactions [218–220], which warrant further investigations into the biodistribution and persistence of immunogen [79, 218]. Additionally, potent type I interferon responses associated with inflammation and autoimmunity have been observed in a few cases [221, 222], and extracellular RNA can potentially result in edema and promote blood coagulation and pathological thrombus formation [223, 224]. Therefore, longitudinal studies are required to monitor and assess the safety profile of these vaccines and to further understand the durability of the immunity provided [225]. Recently, Zhang et al. performed a head-to-head comparison of immune memory and antibody responses in humans from a diverse set of vaccines. The antibody titers were higher for mRNA-1273 and BNT162b2 vaccines than Ad26.COV2.S and NVX-CoV2373. Meanwhile, the memory CD8^+^ T cells showed similar frequencies for both the mRNA vaccines and Ad26.COV2.S. In general, the authors concluded that different types of vaccines induce different qualities and quantities of immune memory cell and antibody responses [226].

Ongoing global research efforts to improve mRNA-based vaccines and therapeutics include studies on: (1) different classes of novel delivery materials, such as lipids, polymers, proteins, peptides and inorganic materials, (2) rational design of mRNA sequences to optimize chemistry, sequence and structure, (3) pharmacokinetics and pharmacodynamics, bio-distribution, kinetics, toxicology and immunogenicity of different mRNA formulations, (4) mRNA manufacturing processes for scalability, cost-effectiveness, purity and stability, and (5) mRNA formulations that can be delivered to different tissues in a safe and effective manner [227]. The most commonly reported severe allergic reaction is anaphylaxis. Several PEGylated drugs have been documented for the cause of allergic effects involving infusion reactions to anaphylaxis [228]. At present, the level of anti-PEG antibodies (APA) remains uncertain to predict the occurrence of allergic responses from the patients with PEGylated lipid-containing mRNA vaccines. It is more practical for clinical setting to remind the patients, pharmacists and physicians to take a precaution of being aware of PEGylated drugs or vaccines. Alternatives to PEG are still under evaluation in clinical trials. Cytotoxicity of lipids is the major safety concern. The secretion of pro-inflammatory cytokines and reactive oxygen species is associated with the use of ionizable lipids in mRNA vaccines. Further investigations in immunogenicity of lipid materials are highly demanded within safety profile.

COVID-19 is known to be a bi-phasic (or multi-phasic) disease, so it requires several different therapeutic options [229]. The first phase of COVID-19 is the viral replication phase; during this phase, drugs that inhibit viral entry or replication can be most helpful. Later, COVID-19 may enter an inflammatory phase in which excessive immune response plays the primary role in damaging the infected individual. Thus, drugs that reduce excessive immune response are most helpful at this stage.

**Fig. 7:**
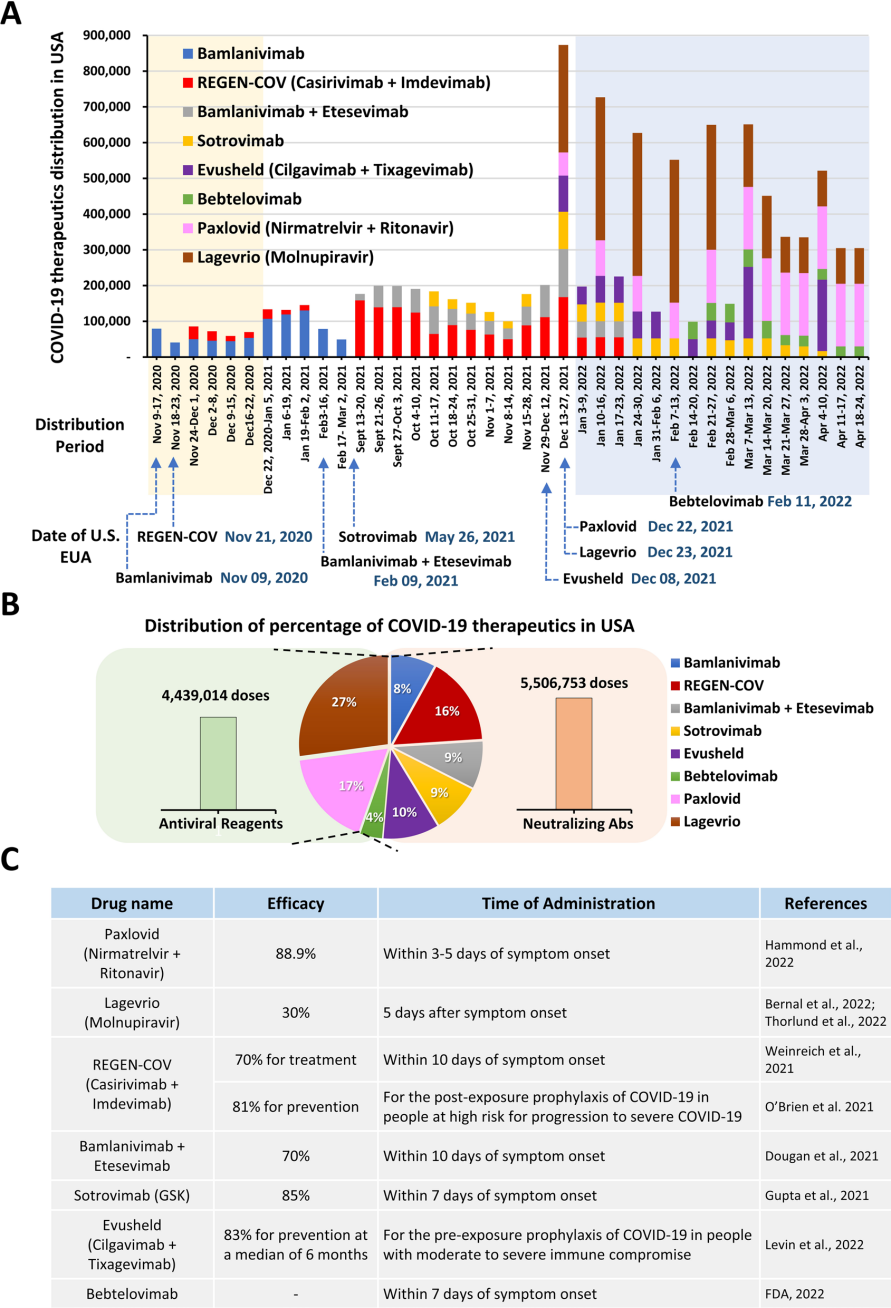
Therapeutics drug distribution and efficacy against COVID-19. **A** Distribution of COVID-19 therapeutics from Nov 9, 2020 to Apr 24, 2022 in USA. **B** Total Distribution percentage of antiviral reagents and neutralizing antibodies doses from Nov 9, 2020 to Apr 24, 2022 in USA. The data was adopted from U.S. Department of Health & Human Service (https://aspr.hhs.gov/COVID-19/Therapeutics/Distribution/Pages/default.aspx). **C** Effectiveness of therapeutic reagents on reducing hospitalization and deaths of COVID-19 patients

**Fig. 8:**
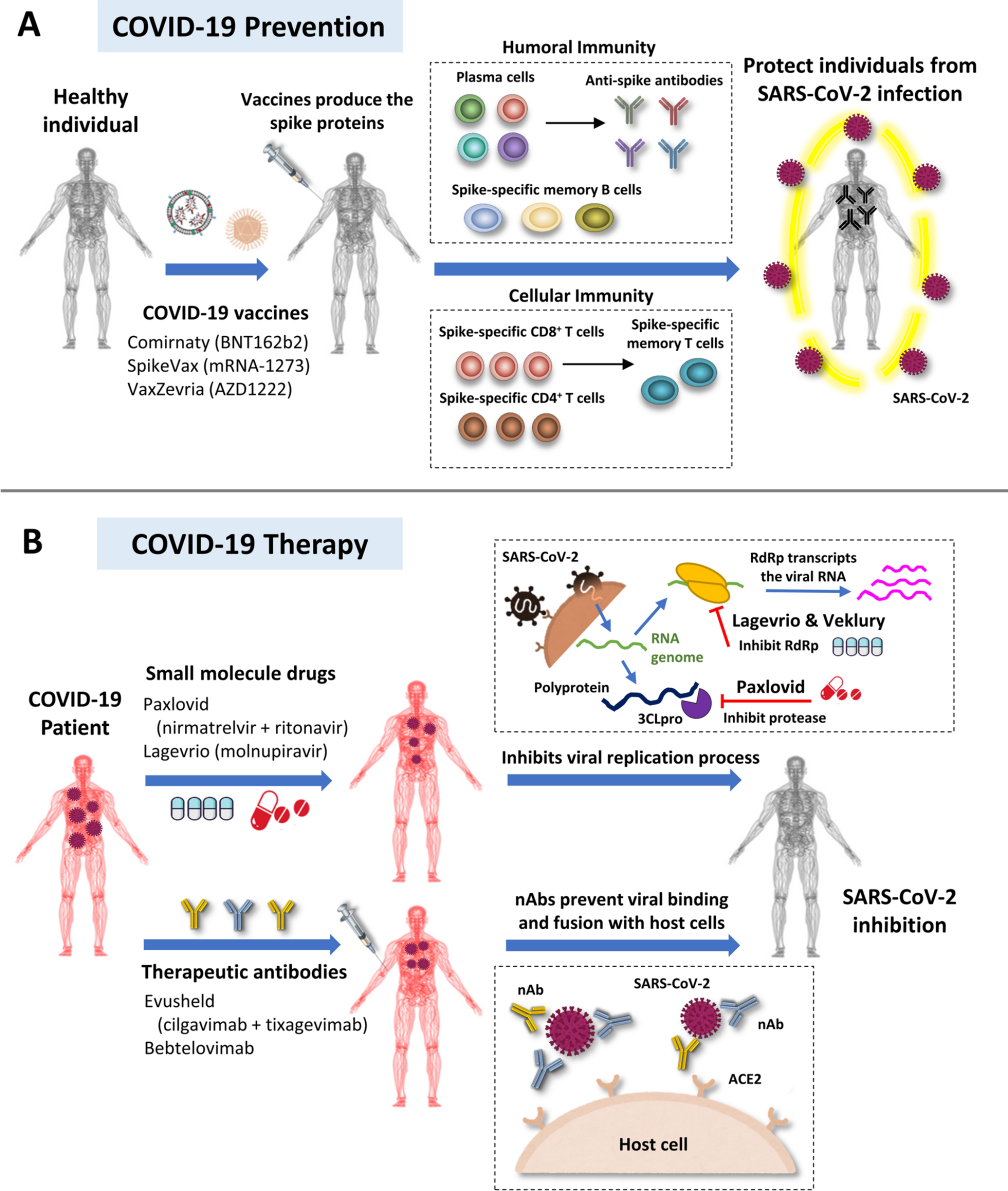
Prevention and therapy for COVID-19. **A** Vaccines stimulate the host immune system to generate neutralizing antibodies against COVID-19. **B** Small molecule drugs and therapeutic antibodies block viral replication or entry

Unsubdued spread of new variants has caused ongoing shortages of COVID-19 drugs, vaccines, and diagnostics in almost all countries. In the US, the government has sought to ensure efficient and fair distribution of COVID-19 therapeutic antibodies and anti-viral drugs by closely monitoring the utilization and coordinating the distribution of COVID-19 therapeutics to hospitals and hospital pharmacies in all states. These measures were implemented with the aim of ensuring prioritized treatment of patients with high risk for severe COVID-19 illness. Figure 7A shows the distributions of eight FDA-authorized COVID-19 therapeutics in the US. Due to the emergence of Omicron as the dominant VOC, the approved use of therapeutic antibodies has been heavily revised, with ineffective antibodies against Omicron removed as treatment options. Allocation of REGEN-COV and bamlanivimab/etesevimab has been paused since January 24, 2022, and allocation of sotrovimab was paused beginning on April 11, 2022. As an alternative to the nAbs, oral anti-viral drugs, such as paxlovid (Pfizer) and lagevrio (Merck), have become the most prominent COVID-19 therapeutics recommended by the US government since the end of 2021 (Figs. 7B, C). These anti-viral agents have clinically proven effectiveness in reducing hospitalization and deaths of COVID-19 patients. The most recent overall anti-COVID-19 strategy is illustrated in Fig. 8.

RBD is the most critical target of anti-SARS-CoV-2 neutralizing antibodies and vaccines. However, widely administration of nAb and vaccination causes the selective pressures to shape SARS-CoV-2 evolution. During the COVID-19 pandemic, the viral variants with RBD mutations, including some residues neighboring the ACE2-binding interface, have continued to arise. The mutations in the RBD not only affect the transmissibility of the virus but also contribute to the escape from host immunity. Therefore, comprehensive analysis of which RBD mutations impact its interaction with ACE2 would aid efforts to understand viral evolution and guide the immunogen design and other countermeasures. Yeast-display platform-based deep mutational scanning approaches have been developed to evaluate how all possible SARS-CoV-2 RBD amino acid mutations affect ACE2-binding affinity [193]. Other techniques such as phage display and pseudoviral RBD libraries also can be applied to perform in vitro evolution experiments to quantify the possible RBD amino acid mutations experimentally. Since SARS-CoV-2 evolution is ongoing, these mutation sites from in vitro selection have opportunity to appear in the RBD of future viral variants. Hence these mutants may be important neutralizing epitopes and provide valuable information on immunogens design for the next-generation vaccines and antibody therapeutics.

Considering that new drug development has been largely impractical during this quickly expanding and changing pandemic, some repurposed or historical drugs (developed for previous diseases) have been authorized or approved for use against COVID-19, including lagevrio (molnupiravir), paxlovid (nirmatrelvir and ritonavir), and Veklury (remdesivir) as well as two potential oral antiviral drugs Sabizabulin (VERU-111) and Ensitrelvir (S-217622, Xocova®) waiting for EUA approval. More importantly, Merck for molnupiravir and Pfizer for paxlovid have entered an agreement with Medicines Patent Pool (MPP), a United Nations-backed public health organization working to expand access to life-saving medicines for low- and middle-income countries on Oct. 27 and Dec. 6, 2021, respectively. Hence, Merck and Pfizer will not receive sale royalties of molnupiravir and paxlovid as long as the WHO continues to classify the pandemic as a global health emergency, respectively. In summary, an ideal drug for treatment of COVID-19 patients should have several essential features, such as diverse blockage mechanisms of virus replication for potential combination use with existing oral antiviral drugs, high efficacy for preventing progression to severe COVID-19 as well as viral rebound, and reliable effects against SARS-CoV-2 variants [230].

The frequent emergence of coronaviruses with high transmissibility or pathogenicity has occurred since the year 2002. Coronaviruses have so far caused the SARS, MERS, and COVID-19 pandemics, which have taken an immense toll on the health and economics of communities around the world. On March 30, 2022, the WHO called for governments to dedicate and invest in strategic plans with the ultimate goal of ending the COVID-19 pandemic in 2022 [231]. To do so, the WHO recommended five key components: (1) boosting surveillance, laboratories and public health intelligence, (2) vaccination, public health, social measures and engaged communities, (3) boosting capacity to treat COVID-19 patients through clinical care for COVID-19 and resilient health systems, (4) prioritizing research and development as well as equitable access to tools and supplies, and (5) ensuring coordination as the response transitions from an emergency mode to long-term respiratory disease management.

As the world recovers from the COVID-19 pandemic, there will remain a need to monitor and treat long-term sequelae in survivors of severe disease. In addition, the further expedited development of vaccines and drugs with broad-spectrum efficacy against existing and future variants will be critical to ultimately overcome current and future challenges associated with the global COVID-19 pandemic.

## Supplementary Information

**Additional file 1. Table S1. **Summarized COVID-19 confirmed total cases, deaths, and death rate in the selected developed countries (up to 08/16/2022). **Table S2. **Current COVID-19 antivirals in development worldwide. **Figure S1.** Chemical structure of EUAs approved small molecule antiviral drugs.

## Data Availability

All the data and materials supporting the conclusions were included in the main paper.
