# Supplementary material for: A critical overview of current progress for COVID-19: development of vaccines, antiviral drugs, and therapeutic antibodies
Source: J Biomed Sci. 2022 Sep 12;29:68. doi: 10.1186/s12929-022-00852-9 (PMC9465653; doi:10.1186/s12929-022-00852-9)
Supplement: Supplementary file 1 — Additional file 1. Table S1. Summarized COVID-19 confirmed total cases, deaths, and death rate in the selected developed countries (up to 08/16/2022). Table S2. Current COVID-19 antivirals in development worldwide. Figure S1. Chemical structure of EUAs approved small molecule antiviral drugs. [file 12929_2022_852_MOESM1_ESM.docx]

**Table S1. Summarized COVID-19 confirmed total cases, deaths, and death rate in the selected developed countries (up to 08/16/2022)**

| **Countries** | **Total cases** | **Total deaths** | **Death rate** |
| --- | --- | --- | --- |
| World | 588,757,628 | 6,433,794 | 1.09% |
| U.S.A. | 91,995,265 | 1,027,845 | 1.12% |
| Canada | 4,109,931 | 43,178 | 1.05% |
| Chile | 4,020,351 | 40,687 | 1.01% |
| Spain | 13,306,301 | 111,906 | 0.84% |
| Italy | 21,554,626 | 174,300 | 0.81% |
| U.K. | 23,461,239 | 187,018 | 0.80% |
| Sweden | 2,551,996 | 19,528 | 0.77% |
| Belgium | 4,460,582 | 32,410 | 0.73% |
| Hong Kong | 1,438,891 | 9,589 | 0.67% |
| Germany | 31,725,160 | 146,214 | 0.46% |
| France | 33,275,006 | 149,848 | 0.45% |
| Switzerland | 3,993,684 | 13,489 | 0.34% |
| Netherlands | 8,370,162 | 22,561 | 0.27% |
| Norway | 1,458,456 | 3,890 | 0.27% |
| Israel | 4,617,144 | 11,503 | 0.25% |
| Japan | 16,161,801 | 35,955 | 0.22% |
| Denmark | 3,259,684 | 6,833 | 0.21% |
| Taiwan | 4,975,970 | 9,540 | 0.19% |
| New Zealand | 1,690,553 | 2,630 | 0.16% |
| Australia | 9,851,002 | 13,021 | 0.13% |
| Republic of Korea | 21,861,296 | 25,813 | 0.12% |
| Singapore | 1,805,698 | 1,567 | 0.09% |

**Table S2. Current COVID-19 antivirals in development worldwide.**

| **Drug name** | **Mechanism of Action (MOA)** | **Delivery** | **Status** | **Company** | **Trial ID** |
| --- | --- | --- | --- | --- | --- |
| **Veklury (Remdesivir)** | RdRp inhibitor | IV | US FDA Approved | Gliead | NCT04280705 NCT04292899 |
| **Olumiant (Baricitinib)** | JAK1/2 inhibitor | Oral | US FDA  Approved | Eli-lilly | NCT04401579 |
| **Lagevrio**  **(Molnupiravir)** | RdRp inhibitor | Oral | EUA | Merck & Co. | NCT04575597 |
| **Paxlovid (Nirmatrelvir + ritonavir)** | 3CL protease inhibitor | Oral | EUA | Pfizer | NCT04960202 |
| **Xocova  (Ensitrelvir)** | 3CL protease inhibitor | Oral | EUA in Japan | Shionogi | NCT05305547 |
| **VV116 (JT001)** | RdRp inhibitor | Oral | Phase III | Shanghai JunTop Biosciences | NCT05242042 |
| **Bemnifosbuvir (AT-527)** | RdRp inhibitor | Oral | Phase III | Atea & Roche | NCT05059080 |
| **Proxalutamide  (GT0918)** | AR antagonist | Oral | Phase III | Suzhou Kintor Pharmaceutical | NCT04869228 |
| **Reparixin** | CXCR1/CXCR2 antagonist | Oral | Phase III | Dompé Farmaceutici | NCT05254990 |
| **C-21** | AT2R agonist | Oral | Phase III | Vicore Pharma | NCT04880642 |
| **Plitidepsin** | eEF1A inhibitor | IV | Phase III | PharmaMar | NCT04784559 |
| **Apabetalone** | BET inhibitor | Oral | Phase III | Resverlogix | NCT04894266 |
| **Carrimycin** | Protein 50S ribosomal subunit inhibitor | Oral | Phase III | Shenyang Tonglian | NCT04672564 |
| **Azvudine** | Nucleoside reverse transcriptase inhibitor | Oral | Phase III | HRH Pharm. | NCT05033145 |
| **Sabizabulin  (VERU-111)** | Microtubular inhibitor | Oral | Phase III | Veru | NCT04842747 |
| **Ivermectin (K-237)** | Host-directed agent | Oral | Phase III | Kowa | NCT05056883 |
| **XC221** | Anti-viral | Oral | Phase III | RSV Therapeutics | NCT04940182 |
| **Nitazoxanide** | Pyruvate: ferredoxin oxidoreductase inhibitor | Oral | Phase III | Romark Laboratories | NCT05157269 |
| **Camostat Mesilate (FOY-305)** | TMPRSS2-related proteases | Oral | Phase III | Ono Pharmaceutical | NCT04657497 |
| **Nafamostat mesilate** | TMPRSS2 inhibitor | IV | Phase III | Katsura Chemical Co. | NCT04390594 |
| **Mesupron**  **(Upamostat)** | TMPRSS2 inhibitor | Oral | Phase III | Red Hill BioPharma | NCT04723537 |

| (A) |
| --- |
| 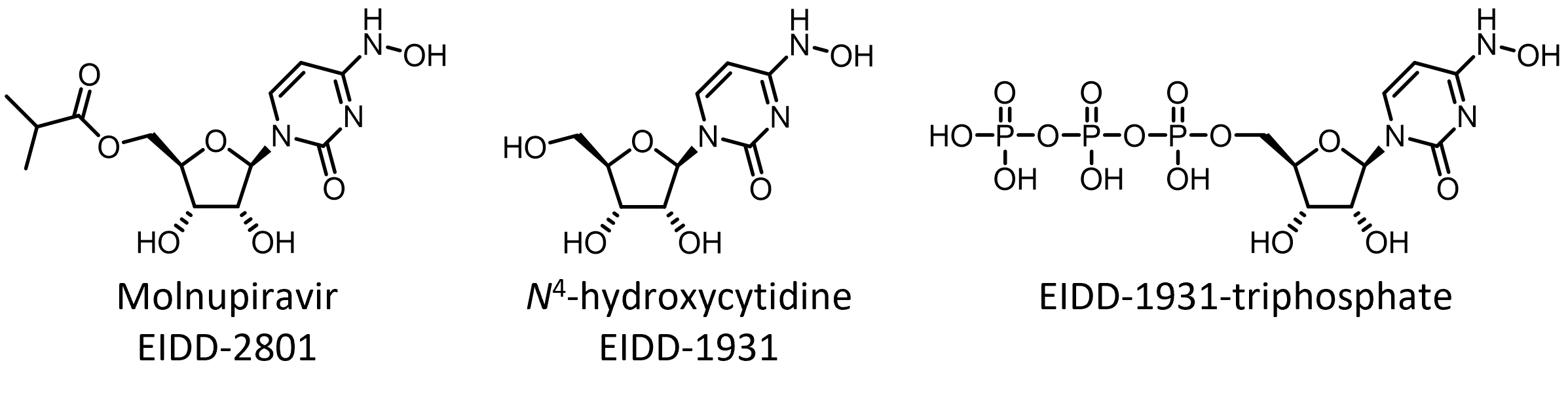 |
| (B) |
| 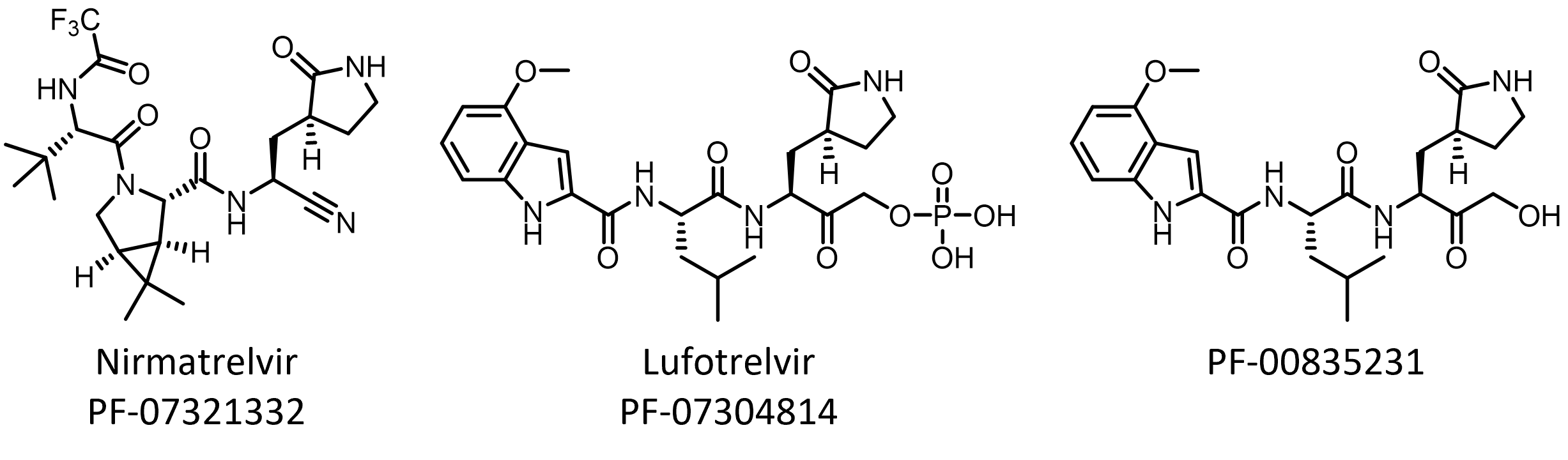 |
| (C) |
| 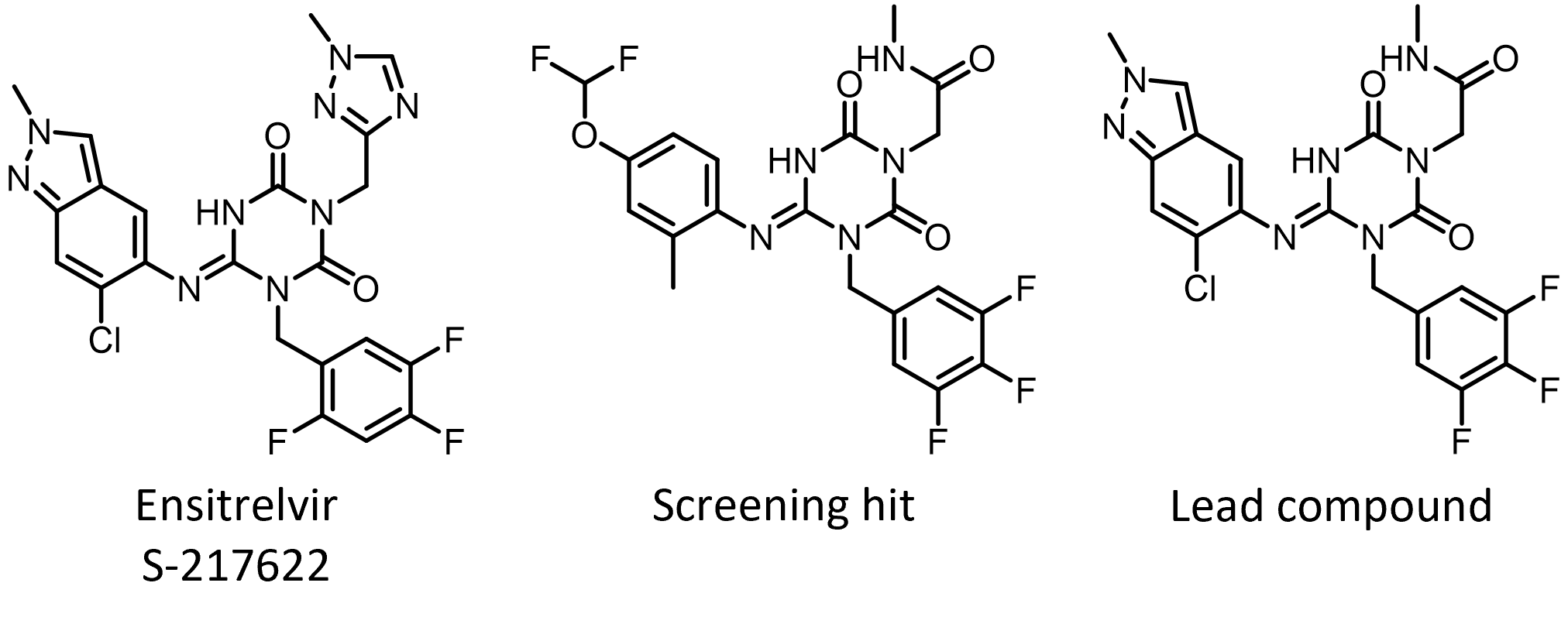 |
| (D) |
| 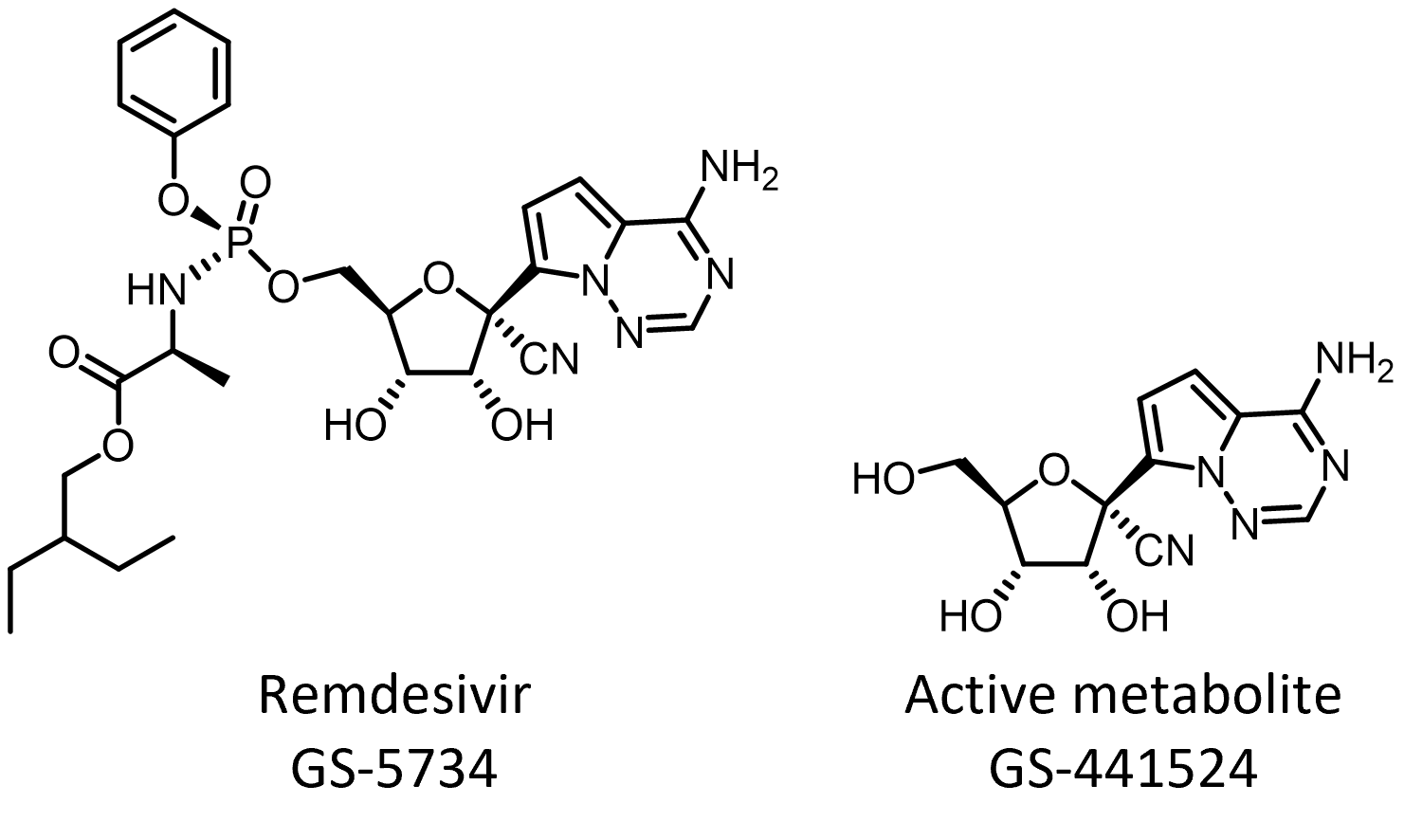 |

**Figure S1. Chemical structure of EUAs approved small molecule antiviral drugs.**
